# The Development of a High Density Linkage Map for Black Tiger Shrimp (*Penaeus monodon*) Based on cSNPs

**DOI:** 10.1371/journal.pone.0085413

**Published:** 2014-01-17

**Authors:** Matthew Baranski, Gopalapillay Gopikrishna, Nicholas A. Robinson, Vinaya Kumar Katneni, Mudagandur S. Shekhar, Jayakani Shanmugakarthik, Sarangapani Jothivel, Chavali Gopal, Pitchaiyappan Ravichandran, Matthew Kent, Mariann Arnyasi, Alphis G. Ponniah

**Affiliations:** 1 Nofima, Ås, Norway; 2 Central Institute of Brackishwater Aquaculture, Raja Annamalai Puram, Chennai, Tamil Nadu, India; 3 Flinders University, Bedford Park, South Australia, Australia; 4 Centre for Integrative Genetics (CIGENE), Department of Animal and Aquacultural Sciences, Norwegian University of Life Sciences, Ås, Norway; National Science and Technology Development Agency, Thailand

## Abstract

Transcriptome sequencing using Illumina RNA-seq was performed on populations of black tiger shrimp from India. Samples were collected from (i) four landing centres around the east coastline (EC) of India, (ii) survivors of a severe WSSV infection during pond culture (SUR) and (iii) the Andaman Islands (AI) in the Bay of Bengal. Equal quantities of purified total RNA from homogenates of hepatopancreas, muscle, nervous tissue, intestinal tract, heart, gonad, gills, pleopod and lymphoid organs were combined to create AI, EC and SUR pools for RNA sequencing. *De novo* transcriptome assembly resulted in 136,223 contigs (minimum size 100 base pairs, bp) with a total length 61 Mb, an average length of 446 bp and an average coverage of 163× across all pools. Approximately 16% of contigs were annotated with BLAST hit information and gene ontology annotations. A total of 473,620 putative SNPs/indels were identified. An Illumina iSelect genotyping array containing 6,000 SNPs was developed and used to genotype 1024 offspring belonging to seven full-sibling families. A total of 3959 SNPs were mapped to 44 linkage groups. The linkage groups consisted of between 16–129 and 13–130 markers, of length between 139–10.8 and 109.1–10.5 cM and with intervals averaging between 1.2 and 0.9 cM for the female and male maps respectively. The female map was 28% longer than the male map (4060 and 2917 cM respectively) with a 1.6 higher recombination rate observed for female compared to male meioses. This approach has substantially increased expressed sequence and DNA marker resources for tiger shrimp and is a useful resource for QTL mapping and association studies for evolutionarily and commercially important traits.

## Background

Crustaceans constitute 10% of the world's aquaculture production and have been the fastest growing aquaculture sector (average growth in production of 15% per year from 1970, reaching 5 million tonnes in 2008) [1]. More than 80% of shrimp exports from India are derived from aquaculture production. One of the most valuable species cultured in India is the black tiger shrimp (*Penaeus monodon*), however aquaculture of this species has been dramatically impacted by outbreaks of disease, in particular white spot syndrome virus (WSSV). Improved disease resistance and growth rate, through both traditional selective breeding practices and applied genomic tools, could have a major positive impact on the sustainability and productivity of *P. monodon* culture in the future. As disease resistance to WSSV infection has low heritability [2], and has been shown in *Litopenaeus vannamei* to be negatively correlated with growth rate [3], the application of genomic selection or marker assisted selection would greatly benefit the genetic improvement possible for this particular trait. Reproductive performance traits such as multiple spawning capacity are also of importance to *P. monodon* aquaculture and applied genomic tools have potential for advancing our comprehension and the genetic improvement of such traits [4].

Relatively speaking, there is a paucity of genomic resources for *P. monodon* [5]. *P. monodon* has 44 chromosomes and a genome size of ∼2.17×10^9^ bp [6]. In GenBank there are 39,908 expressed sequence tags that could potentially be used to detect polymorphic loci (such as single nucleotide polymorphisms or SNPs) and around 600 microsatellite sequences (available 1 October 2013) [7]–[20]. The most recent linkage map for *P. monodon* consists of 256 microsatellites and 85 Amplified Fragment Length Polymorphism (AFLP) markers [6]. Microsatellites have been commonly used in earlier linkage mapping efforts in *P. monodon*, as they are naturally abundant, codominant and highly informative [8], [11], [15], [18], while AFLPs have been commonly used because they are easily generated without prior knowledge of genomic sequence [6], [21]–[25].

However, recent advances in SNP genotyping technology have made it more cost effective and efficient to genotype several thousand SNP markers simultaneously on a single array rather than a large number of microsatellite multiplexes or AFLPs. Furthermore, if SNPs can be selected to give comprehensive genome coverage this will increase the likelihood of finding loci in close linkage disequilibrium (LD) to causative genes (ie. Quantitative Trait Loci: QTL) as well as facilitate fine mapping and verification of known QTL. For instance, a gene homologous to the elongation of very long fatty acids-like (ELOVL) protein family has been proposed as a candidate gene affecting growth rate in crustaceans [26]. Such genes could be verified and fine-mapped using a panel of densely mapping transcribed SNPs.

Here we use Illumina RNA-seq to characterise the transcriptomes of *P. monodon* from populations along the coast of India and further afield in the Andaman Islands, along with a population of tiger shrimp that survived a very heavy infection in a farmer's pond in Andhra Pradesh. The transcriptome data generated was used to identify a large number of putative SNPs and indels within transcribed genes, and a high-density genetic map containing around 4000 of these SNPs was produced using a custom Illumina iSelect genotyping array.

## Results

### *De-novo* assembly

**Figure 1:**
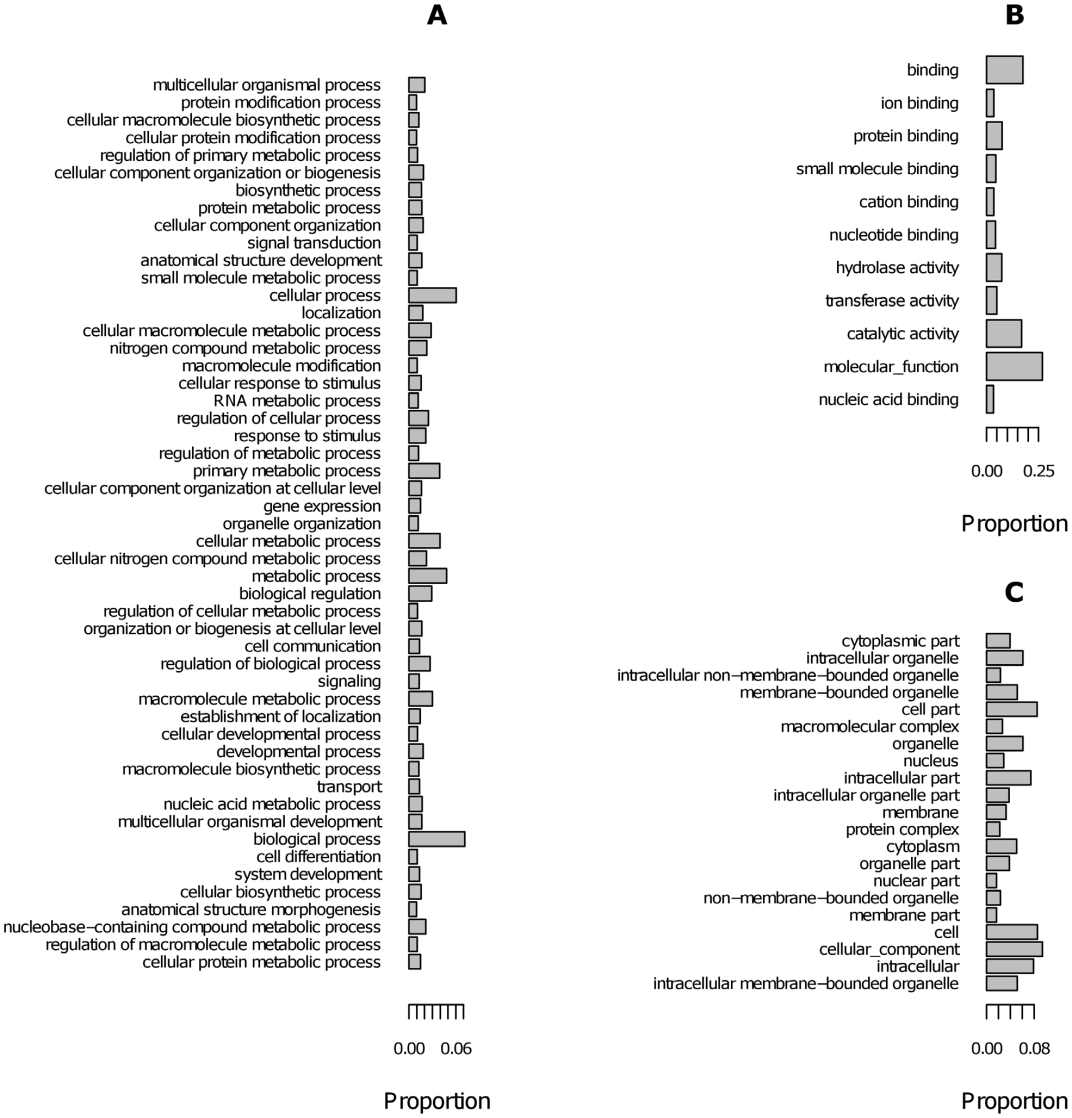
Proportion of gene ontology terms related to (A) process, (B) function and (C) category, for transcripts containing linkage mapped SNPs.

In total, 196 million reads containing 14 billion bases were assembled into 136,223 *de novo* contigs containing around 61 million bp of sequence with an average contig length of 446 bp (Table 1). Alignment of the reads to the *de novo* assembly, revealed the average coverage to be 163×, while the coverage within each lane (population) ranged from 48–64×. Roughly 16% of contigs could be annotated with BLAST hit information. The majority of the top BLAST hits (>2000) were against the red flour beetle (*Tribolium castaneum*), with fewer than 500 contigs displaying top hits to *P. monodon* sequence, which reflects the low numbers of crustacean sequences lodged in GenBank. A range of biological processes, functions, categories and pathways were represented by the transcribed genes containing mapped SNPs (Figure 1). A high proportion of sequences were categorised as having binding (eg. protein binding) or catalytic activity (eg. hydrolase activity) functions (Figure 1B). The longest contig had a length of 23,796 bp (contig 52374 with 77% identity to projectin from freshwater crayfish *Procambarus clarkia*). When the minimum contig size was set to 200 bp, the total number of contigs was reduced to 52,106, with an average of length of 950 bp.

**Table 1:** Summary of results from Illumina GAII sequencing performed on cDNA pools.

	AI^1^	EC	SUR	All
**Before trimming**				
Number of reads	77.7 million	59.2 million	59.4 million	196.3 million
Number of bases	5.60 Gb	4.26 Gb	4.28 Gb	14.14 Gb
Average read length	72	72	72	72
**After trimming**				
Number of reads	68.3 million	52.5 million	52.7 million	173.6 million
Number of bases	4.59 Gb	3.54 Gb	3.57 Gb	11.70 Gb
Average read length	67	67	68	67
**Number of contigs**				136,223 (52,106)
Total sites				60.7 Mb (49.5 Mb)
Average contig length				446 (950)
Longest contig				23,796
No. assembled reads	58.4 million	43.8 million	45.2 million	147.3 million
Average coverage	64	48	50	163

Results are shown before and after adaptor, poly-A trimming and quality filtering. *De novo* transcriptome assembly was performed using the combined dataset (All). Results of the ‘mapping assemblies’ for the detection of SNPs are also shown. *De novo* contig assembly statistics are also shown in parentheses for more stringent settings (minimum contig size of 200 bp).

^1^ AI, EC and SUR represent sequence results for individual RNA pools made of samples from the Andaman Island landing centre, East Coast landing centres (Chennai, Vishakhapatnam and Tuticorin) and surviving animals from a WSSV outbreak on a farm in Bapatla respectively.

### Single nucleotide polymorphisms

A total of 473,620 SNPs and indels were identified at the most relaxed detection threshold of which 20,372 had minor allele frequencies ≥0.3 and coverage ≥100× (Table 2). Six thousand SNPs were selected for the creation of the Illumina iSelect SNP array (Dataset S1). Sixty-two percent of these SNPs could be annotated with gene names leaving 38% with unknown identity. Genotyping using the Illumina iSelect SNP array revealed that 4237 SNPs could be clustered and were polymorphic in the mapping families.

**Table 2:** Single nucleotide polymorphism detection statistics (includes insertions/deletions).

Parameter	Range	Number of SNPs
**Coverage (Cov)**	≥1000×	11,820
	≥100×	132,859
	≥50×	242,453
	≥25×	359,054
**Minor allele frequency (MAF)**	≥0.4	50,612
	≥0.3	114,956
	≥0.2	204,708
	≥0.1	345,740
**Coverage and minor allele frequency**	≥1000× and ≥0.4	747
	≥100× and ≥0.3	20,372
	≥50× and ≥0.2	70,076
	≥25× and ≥0.1	231,948
**Total number of putative SNPs detected**	Cov ≥6, MAF ≥0.05, Min allele ≥3^1^	473,620

^1^ Minimum count of non-reference alleles.

### Microsatellite discovery

A total of 7207 microsatellites were detected within 6492 contigs, 3% were mono-nucleotide repeats, 65% di-, 24% tri-, 5% tetra-, 1% penta- and 2% hexa-nucleotide repeats, Dataset S2). Candidate PCR primers could be designed for 1506 of these (Dataset S3). The candidate microsatellite markers identified were not used for the pedigree assignment or linkage mapping described below.

### *P. monodon* mitochondrial transcriptome

**Figure 2:**
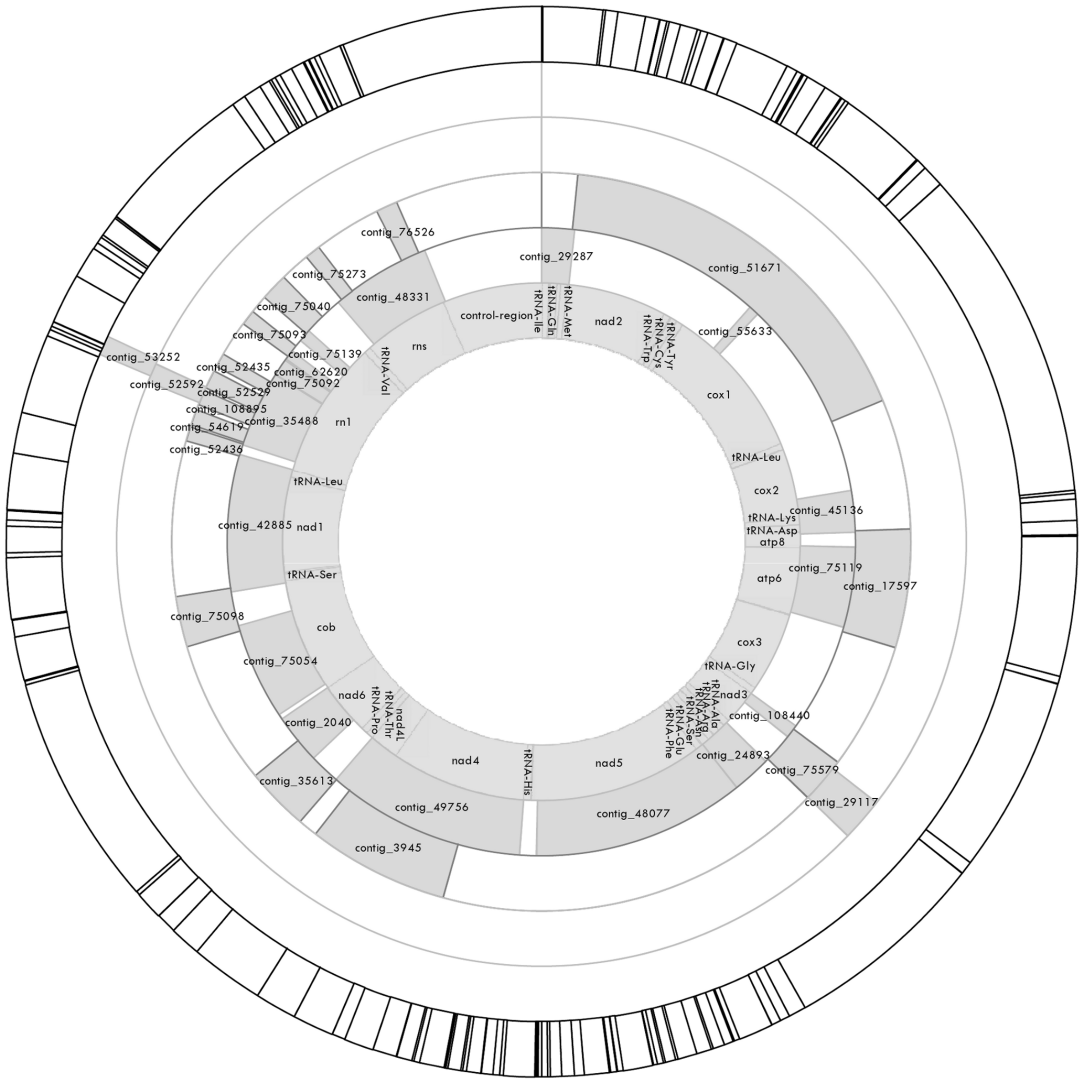
Map of the *P. monodon* mitochondrial genome. The inner ring shows the location of mitochondrial genes, t-RNA and the control region. The position of assembled contigs are represented by shaded patches in the four concentric rings out from the inner ring (labelled contig IDs) and SNP positions are marked by lines in the outer ring. A double line dividing the top of the outer ring marks the start position for the *P. monodon* mitochondrial DNA sequence.

Two hundred and seventy three contigs were annotated with the word “mitochondria” and of these 34 were found to map to the *P. monodon* mitochondrial DNA sequence in GenBank (GenBank AF217843.1). All 15 protein coding regions of the mitochondria were expressed in the tissues sampled. In these 34 contigs, 145 SNPs were detected (Dataset S4 and Figure 2).

### Genomic pedigree assignment

Initial assessment of Mendelian inheritance identified possible errors in the documented pedigree. Pairwise relatedness clustering of 4237 SNPs identified seven distinct clusters of individuals representing full-sibling families, each containing offspring and a sire, dam or both parents. Offspring that did not fall within the identified full-sibling families and genotypes that were not inherited in a Mendelian fashion (eg. due to clustering errors) were removed from the analysis. After recreation of missing parental genotypes, the final SNP dataset for linkage mapping consisted of seven full-sibling families (1–7) with a total of 1024 offspring (266, 138, 124, 124, 123, 123 and 126 in each full-sibling group respectively).

### Linkage map

At an initial LOD threshold of 20, 42 linkage groups were identified. Increasing the LOD threshold to 60 resulted in the splitting of two of these groups into two smaller groups, forming 44 linkage groups that matched the expected haploid chromosome number in *P. monodon*. In all, 3959 informative SNP markers mapped to these 44 linkage groups (Figure S1).

The female and male maps contained 3592 and 3486 SNP markers respectively and 3124 SNP markers were informative for both male and female maps. The female linkage map covered 4060 cM with an average interval of 1.2 cM and a maximum interval of 32.8 cM. The length of the linkage groups ranged from 139.3 cM to 10.8 cM and the number of markers varied from 129 to 16 per group (Table 3). The genome size estimate for the female was 4170.9 cM resulting in 82% coverage of the genome within 1 cM of a framework marker.

**Table 3:** Comparison of map intervals and number of SNPs between male and female maps.

	Female			Male			Sex-average		
LG^1^	Avg (cM)	Max (cM)	SNPs	Avg (cM)	Max (cM)	SNPs	Avg (cM)	Max (cM)	SNPs
1	2,0	17,8	44	1,9	17,8	42	1,5	14,5	48
2	1,4	11,3	79	1,1	9,0	77	1,0	10,1	91
3	1,1	9,2	66	0,7	9,3	68	0,9	9,2	70
4	1,1	13,0	83	0,7	9,8	84	0,8	8,4	95
5	1,1	11,5	101	0,9	10,0	87	0,9	8,8	110
6	1,2	18,1	92	0,9	8,8	87	0,9	11,7	97
7	1,1	11,0	97	0,8	12,9	91	0,9	9,0	102
8	1,1	12,1	93	0,8	8,4	87	0,8	7,3	102
9	0,9	19,5	125	1,0	15,5	114	0,8	14,2	134
10	0,9	15,4	119	0,8	9,4	113	0,8	8,6	131
11	1,3	14,5	54	1,1	7,8	57	1,0	10,4	61
12	1,8	12,5	41	1,3	8,9	40	1,5	10,8	45
13	1,2	32,1	65	0,8	18,9	70	0,9	25,8	76
14	0,8	17,4	129	0,7	12,3	127	0,7	14,9	140
15	1,4	14,5	60	0,9	7,9	61	1,1	9,1	64
16	1,0	11,1	84	0,6	6,8	89	0,7	8,2	94
17	1,3	14,4	62	0,9	5,7	61	0,9	8,1	72
18	1,6	13,4	65	1,0	9,8	68	1,1	11,9	75
19	0,9	17,6	129	0,6	6,8	130	0,7	11,4	141
20	1,1	10,6	107	1,0	10,8	102	0,9	9,3	125
21	1,1	9,6	94	0,9	9,5	88	0,9	8,9	99
22	1,3	32,6	97	0,8	10,6	92	1,0	22,1	103
23	1,0	20,4	104	0,7	10,2	107	0,7	8,4	122
24	0,9	17,8	90	0,7	12,2	78	0,7	15,2	95
25	0,9	7,2	93	0,7	6,7	94	0,7	5,3	104
26	1,3	19,5	84	1,1	8,0	81	1,1	12,7	89
27	1,7	32,8	85	1,1	7,0	79	1,2	11,9	91
28	1,0	16,2	107	0,6	4,7	97	0,7	7,4	118
29	1,1	12,8	100	1,0	13,3	88	0,9	11,4	110
30	1,3	19,2	80	0,8	7,6	84	0,9	13,3	92
31	1,4	11,4	54	1,0	12,7	54	1,2	11,3	59
32	0,9	11,9	115	0,7	6,9	101	0,7	9,3	125
33	1,0	12,0	76	0,7	5,1	82	0,8	7,9	87
34	0,9	8,9	93	0,7	7,8	90	0,7	8,3	101
35	1,1	8,5	60	1,0	11,9	57	0,9	6,5	67
36	1,2	16,0	77	0,7	8,7	81	0,9	13,3	86
37	1,2	11,8	61	0,8	8,7	63	0,9	7,8	71
38	2,1	20,3	49	0,9	7,4	61	1,2	12,5	64
39	1,4	10,4	59	1,0	6,8	55	1,1	7,2	62
40	2,4	17,3	30	1,9	22,6	32	2,0	20,3	35
41	1,0	14,5	87	0,7	6,2	80	0,8	9,5	93
42	1,0	13,4	85	0,7	8,3	79	0,8	9,7	89
43	0,7	2,8	16	0,9	7,7	13	0,7	3,6	17
44	1,2	26,3	101	0,7	4,7	95	0,9	10,8	107
**TOTAL**			3592			3486			3959

^1^ LG, linkage group.

The male linkage map covered 2917 cM with an average interval of 0.9 cM and a maximum interval of 22.6 cM. The length of the linkage groups ranged from 109.1 cM to 10.5 cM and the number of markers varied from 130 to 13 per group. The genome size estimate for the male was 2997.7 cM resulting in 91% coverage of the genome within 1 cM of a framework marker.

The female map was 28% longer overall than the male map. Female recombination frequency was on average 1.6 times greater than the rate in male meiosis over all the linkage groups (*P*<0.001). All female linkage groups were longer and had longer map intervals between markers than male linkage groups (t-tests of map intervals approaching significance for 13 linkage groups with *P*<0.05 and 11 with *P*<0.01 without Bonferroni correction).

Approximately 1% of markers did not segregate according to Mendelian expectations in some families following Bonferroni correction based on the number of linkage groups (*P*<0.05, Dataset S7).

### Position of selected genes on linkage map

Fifty-nine percent of SNPs were annotated with gene names (Karlin-Altshul cut off E-score of 0.001) and 34% with GO terms (Datasets S5 and S6). Some gene homologies of interest from an immune perspective included toll receptor and pathway genes (positioned at 63.6 cM on linkage group 7 and at 43 cM on LG 35), heat shock proteins and interacting genes (heat shock protein 70 interacting protein, heat shock protein 40, 70, isoform a, 21, 67b2, 83 and 10kd protein1 which were positioned at 47.6, 79.1 and 82.7 cM on LG14; 37.8 and 38.4 cM on LG19; 88.5 and 104 cM on LG20; and, 28 cM on LG26 respectively), 11 lectins (found on linkage groups 16, 20, 23, 24, 31, 34, 35, 42 and 43) and 34 ubiquitin associated protein ligases, carboxy-terminal hydrolases, domains, interacting proteins, specific proteases, conjugation enzymes and factors, specific pepsidases and fold modifiers (found on linkage groups 4–7, 9, 11, 13, 15, 17–19, 21, 23, 24, 26, 31, 32, 34, 37 and 38). Animals surviving 84 hours post-infection with White Spot Syndrome Virus (WSSV) have higher expression of lysozyme, C-type lectin, penaeidins, prophenoloxidase-1 and prophenoloxidase-2 in haemocytes than those dying less than 60 hours post-infection [27]. Contigs with homology to lysozymes and penaedins were not linkage mapped in our study, but contigs with homology to c-type lectins were found to map to positions 66.5 cM on LG16, 17.8 and 18.1 cM on LG42 and to 2.2 cM on LG43, c-type lectin 4 to position 43.2 cM on LG23 and c-type lectin 5 to 43.4 cM on LG42, prophenoloxidae-b to 78.9 cM on LG27 and prophenoloxidase activating enzyme 2a to 36.9 cM on LG36. Heat shock protein 21 is down regulated after infection to WSSV and was found to map to 38.4 cM along LG19 [28].

## Discussion

This study has generated an extensive new resource of genetic markers (SNPs and microsatellites), transcribed gene sequences, and a genetic linkage map for *P. monodon*. These resources will likely prove to be useful for population genetic studies, gene expression analyses and mapping studies to find QTL for commercially and evolutionarily important traits like disease resistance. The linkage map produced here represents the most comprehensive genetic map published for any shrimp species to date.

Samples were taken from different tissues chosen from a number of individuals sourced from different populations in India in a deliberate effort to capture as much genetic variability within expressed genes as possible and thereby ensure that a large number of polymorphisms would be available for genetic marker development. In the absence of a reference genome sequence for a species, transcriptome sequencing (RNA-seq) is a good option for *de novo* gene and marker discovery requiring a relatively small amount of sequencing eg. [29]. While there exists an EST resource for this species in Genbank, no reference genome data is available, hence it was decided to perform a *de novo* assembly to ensure that novel transcripts were represented as a possible source of genes and markers. *De novo* transcriptome assembly is complicated by the presence of splice variants and other structural variations. The increasing read lengths provided by NGS sequencing technologies and improvements being made to assembly software, help to generate more accurate assemblies and enable the detection of structural variants. *De novo* transcriptome assemblies have greatly increased the available expressed sequence for other species that lack reference genomes eg. [29]–[32]

NGS technologies are beginning to be used as a means for performing an initial scan of the genome for a variety of organisms for the study of the genetic basis of local adaptation and speciation eg. [33] and to detect associations with quantitative trait loci eg. [34]. SNP allele frequencies or gene expression values between experimental groups or locally adapted populations can be compared by sequencing multiple samples or pools, through physical separation between flow cells or through multiplexing with sequence barcodes. Variation in RNA amounts among individuals has not been found to be a serious problem in previous studies [33] and can be controlled, and/or normalised, depending on the methodology used. By adopting such approaches it is possible to discriminate between different experimental groups or populations rapidly and economically using a fraction of the capacity of instruments such as the Illumina HiSeq 2000. This promises to provide a powerful means to scan the genome if phenotypic differences between experimental groups or populations can be clearly defined, and if other factors influencing the expression of the trait can be experimentally controlled. Our primary aim was to use the technology to detect and map informative, gene-oriented SNPs for subsequent studies. Although we were unable to set up rigorous experimental comparisons between sequenced shrimp, differences between the groups of animals sequenced, and the baseline data from this study, will be of general interest and use to the *P. monodon* research community.

### Differences between individuals sequenced

In this study a SNP/indel density of one polymorphism for every 128 bp was found when the most ‘relaxed’ SNP/indel detection threshold was used. This density was reduced to one polymorphism for every 261 bp using more stringent SNP/indel detection criteria (min allele freq. 0.1 and min coverage 25). This is similar to the finding of one SNP every 285 bp reported for ESTs in *L. vannamei* [35]. In abalone, EST-SNP densities have been reported to be as high as 1 SNP per 100 bp [36]. The SNP density in individual genes and contigs can be used to make inferences about the level of conservation and selection pressure on these genes. Highly conserved genes are characterised by the occurrence of few SNPs in long, highly sequenced contigs [29]. In any case, SNP/indel detection results are highly dependent on the stringency of thresholds applied. In our study, the number of detected polymorphisms ranged from tens to hundreds of thousands depending on the detection thresholds, and in the literature such thresholds are rarely consistent between studies. If informative genotyping assays are desired, then more stringent thresholds are likely to improve the rate of success by avoiding sequence errors and other artefacts. Splice variants and intron/exon boundaries can also affect the success of genotyping assays and for the former it has been suggested that a high density of SNPs in a short distance can be indicative of such events in sequence assemblies eg. [37]. Access to a well annotated reference genome enhances the ability to differentiate true SNPs from ‘false positives’ in the form of structural variants, and other ‘problematic’ regions (such as intron-exon boundaries).

Our detection of abundant microsatellite loci in the expressed genome mirrors the findings from previous studies of the *P. monodon* genome [11], [20]. Although the candidate microsatellite loci identified in the transcriptome were not genotyped or mapped in this study, the loci could be useful markers for supplementing future genome scans for quantitative trait loci, for population studies or pedigree analysis. The *P. monodon* genome has been found to contain a higher abundance of microsatellite loci than that of vertebrates, and is 4× higher than that in the *Fugu* genome. Sequencing of a *P. monodon* fosmid library has shown that around fifty percent of the fosmid-ends sequenced consisted of retrotransposons, DNA transposons, WSSV-penaeid repetitive elements, other penaeid repetitive elements or microsatellites [20].

The reason for the high abundance of microsatellites and transposable elements in expressed penaeid genomes is not well understood but it has been hypothesised that the microsatellites could sometimes have conserved functional roles [20]. It will therefore be interesting to examine the repeat length variation in expressed microsatellite loci mapping closely to quantitative trait loci detected by genome scans as some may be in linkage disequilibrium with the causative genetic loci, or represent the causative variants themselves, affecting particular traits. The high abundance of these loci may have resulted in fragmentation with the assembly of the transcribed shrimp genome. Many contigs were found to terminate with microsatellite sequence and it is possible that some repeated sequence has collapsed when contigs with high coverage were assembled.

### *P. monodon* mtDNA transciptome

Figure 2 highlights the fact that the *de novo* assembly performed in this study was not optimal, and led to a certain degree of contig redundancy. For instance, contig 35488, which covers part of the mtDNA rn1 gene, shared overlap with seven smaller contigs, such that contig coverage was four-fold in one area. Longer read lengths and improving algorithms for *de novo* transcriptome assembly would be expected to considerably improve such assemblies, if they allow for the occurrence of repetitive sequence and for ‘transcriptome specific’ issues such as differential splicing.

Our study demonstrates the potential power of RNA-seq for informing genetic studies about patterns of nuclear and mtDNA evolution and variability within and between natural populations. The sequence derived from RNA-seq covered nearly the entire mitochondrial genome apart from areas of MT-CO2, MT-CO3 and the control region (the latter of which is highly variable and untranscribed). Average sequence coverage for contig_45136, representing part of MT-CO2, tRNA-Lys, tRNA-Asp and MT-ATP8, was high (approximately 9000 in AI and 8000X in EC). The SNP coverage was irregular, with an absence of SNPs in long stretches of the MT-CO1, MT-ATP6 and COB6 genes for instance, indicating that these regions are likely to be highly conserved.

### Linkage groups and genome coverage

This study presents a dense genetic linkage map for *P. monodon* containing 3959 SNPs mapped to 44 linkage groups, corresponding to the haploid chromosome number in this species. Total lengths for the male and female maps were 4059 cM and 2917 cM respectively. Other linkage mapping studies for *P. monodon*, Pacific white shrimp *Litopenaeus vannamei*, Chinese white shrimp *Fenneropaeneus chinensis* and Kuruma shrimp *Penaeus japonicus* have estimated much shorter male and female map lengths (Table 4). However, it is generally observed that the more markers used, the longer the estimated map length. The calculation of “genome length” compensates for the number of markers by adding 2 times the average framework marker spacing to the length of each linkage group. With fewer markers there will be a larger compensation. Our genome length estimates (2997.7 cM male and 4170.9 cM female), are almost the same for the male map, but larger for the female map, when compared to those for *P. monodon* made using fewer markers and fewer informative meiosis (estimated male and female genome lengths of 2994 and 3433 cM respectively) [6]. As the haploid chromosome number in *P. monodon* is n = 44 [6], markers are spread over many more linkage groups, and many more markers are needed to give the same level of coverage per chromosome, than is the case for other species with fewer chromosomes. Calculations based on flow cytometry estimates of genome size (2.17×10^9^ bp) and genetic distance have estimated that there are approximately 395 kb per cM distance in the *P. monodon* genome [6].

**Table 4:** Comparison of shrimp linkage map studies.

Species	Marker type	Map length (cM) (number of markers in parentheses)		Difference in ♂ map length	Average distance between markers (cM)		Number of families	Number of offspring	Reference
		♂	♀		♂	♀			
***P. monodon***	SNP	2917 (3486)	4060 (3592)	−28%	0.9	1.2	7	1024	*this study*
	Microsatellite and AFLP	2033.4 (225)	2182.0 (207)	−7%	11.2	13.8	3	413	[6]
	AFLP	2378 (757)	2362 (494)	+1%	3.3	5.2	3	345	[21]
	Microsatellite and EST	1101.0 (157)	891.4 (111)	+19%	7.0	8.0	1	41	[11]
	AFLP	Within families	Within families	Within families	Within families	Within families	3	18	[25]
***Fenneropenaeus chinensis***	AFLP	1617 (144)	1090 (103)	+33%	16.4	14.5	?	?	[38]
	AFLP	1737.3 (194)	2191.1 (197)	−21%	11.0	13.5	2	100	[22]
	Microsatellite and RAPD	1144.6 (46)	1173 (49)	−2%	12.05	11.28	1	82	[58]
***Litopenaeus vannamei***	Microsatellite and AFLP	3220.9 (267)	4134.4 (319)	−22%	?	?	1	94	[39]
	AFLP	2116 (182)	2771 (212)	−24%	?	?	4	40	[40]
	SNP	2130 (418)	2071 (418)	+3%	?	?	3	144	[59]
***Penaeus japonicus***	AFLP	1780 (217)	1026 (125)	+42%	8.3	9.2	1	112	[24]

Pairwise recombination rates between informative linked markers were 1.6 higher in female compared to male meiosis. Large differences in linkage group recombination rates between sexes (eg. 21–42%) have been observed for other crustacean species such as *Fenneropaeneus chinensis* (female map 21% longer than male map in one study and 33% shorter that the male map in another study) [22], [38], *Penaeus japonicas* (male map 42% longer than female map) [24] and *Litopenaeus vannamei* (female map 22–24% longer than male map) [39], [40] (Table 4). More markers were mapped to the female than the male map for our, and one other *P. monodon* study [6]. Estimation of the male to female map length in *P. mondon* has been varied. Maneeruttanarungroj et al. [11] found that the male map was 19% longer than the female map, You et al. [6] found that the female map was 7% longer than the male map, Staelens et al. [21] found that out of three families analysed, the male map was +21, +21 and −8% the length of the female map, and Wilson et al. [25] found that out of three families analysed, the male map was +4, +9 and −31% the length of the female map. So while You et al. [6] and two out of six families in two other studies [21], [25] support our finding that the female map is longer than the male map, Maneeruttanarungroj et al. [11], and four out of six families in two other studies [21], [25], have found the reverse effect. We contend that our study provides more accurate estimates than the previous *P. monodon* studies as many more markers, families, offspring, and therefore many more total informative meioses, were used to create the map (Table 4).

The difference in the female to male map length was found to be consistent across most linkage groups in our study, but was not as large as reported in some organisms such as Atlantic salmon where the ratio of male to female recombination was as high as 8.26 [41]. However, in Atlantic salmon a more recent linkage map found the difference in sex specific recombination rate (1.38∶1) to be much lower than this previously reported value [42]. The sexes displayed striking differences in the distribution of recombination sites within linkage groups, with males showing recombination strongly localized to telomeres. This demonstrates that unless there is sufficient marker coverage across the entire length of all chromosomes, comparisons of male and female recombination rates can be heavily biased. Sex-based recombination differences have implications for both QTL genome scan and marker assisted selection strategies, especially in cases where the differences are extreme. For example, earlier studies in Atlantic salmon utilised as few as two markers per chromosome for a male parent based QTL linkage analysis, followed by fine mapping in female parents using a larger numbers of markers [43]. However, now that efficient genotyping of thousands of markers with SNP arrays or through NGS (eg. RAD-tags) is feasible, such a two-stage strategy may no longer be needed and both coarse and fine mapping of QTL can be performed simultaneously.

## Conclusions

In summary, this study has greatly increased genomic resources for *P. monodon* research through the use of comprehensive transcriptome sequencing and the development of a linkage map containing nearly 4000 SNPs in transcribed genes. The SNPs were identified in a diverse set of samples from populations in the Andaman Islands, the East coast of India and from animals which had survived a severe outbreak of WSSV on mainland India. By sequencing the transcriptomes of animals from a wide variety of sources, including survivors of a WSSV outbreak, there is a chance that some of the variation detected is causative variation, or closely linked to other mutations affecting important traits such as disease resistance or reproductive performance. The mapped SNPs from this study will therefore be a useful resource for scanning the tiger shrimp genome for markers affecting economically important traits for aquaculture. Marker assisted selection eg. [44] using SNPs closely linked to QTL affecting these traits, or genomic selection [45], [46] using the complete set of SNPs identified in this study, could speed the rate of genetic improvement for such traits in the future. The SNP resource will also be useful for fine mapping QTL that are identified using other resources. The results highlight the potential usefulness of RNA-seq using next generation sequencing technologies for SNP identification and for functional annotation of the genome of species for which a full genome reference sequence is unavailable. However, the lack of well-annotated genome sequences for most aquaculture species, including crustaceans, is limiting our ability to study structural variation which could have important influences on traits such as disease resistance. RNA-seq is only one of a number of technologies that needs to be applied to enhance our knowledge about the genetics of these complex traits.

## Methods

### Shrimp tissues sampled for RNA-seq

**Figure 3:**
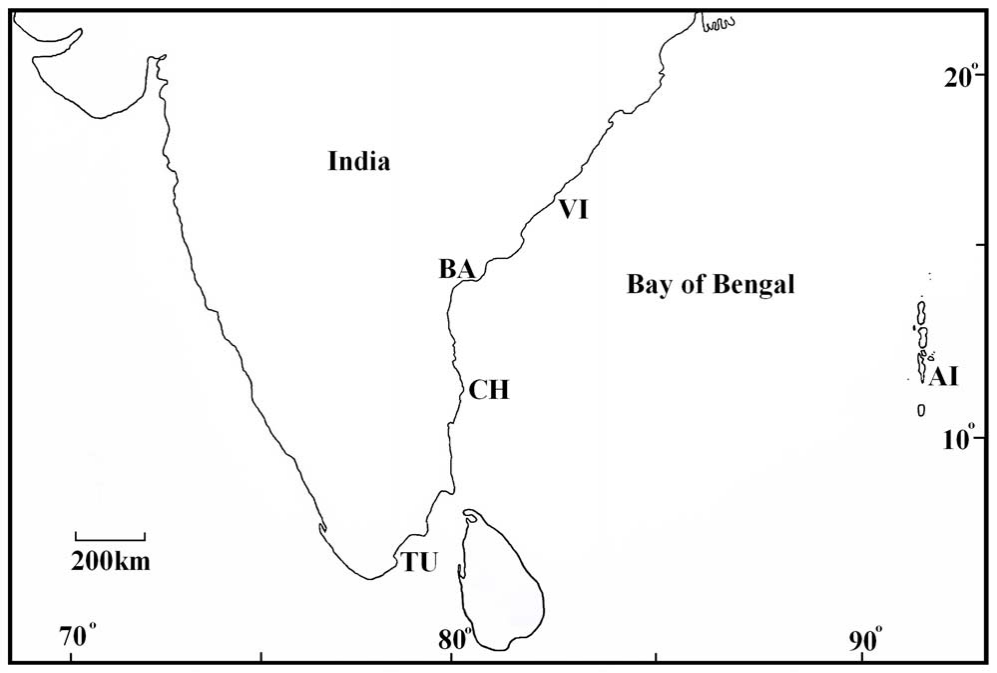
Map showing areas from which *P. monodon* were sampled. *AI*, *CH*, *TU* and *VI* represent catch areas covered by landing centres in the Andaman Islands, Chennai, Tuticorin and Vishakhapatnam respectively. *BA* represents the Bapatla location where shrimp that survived a severe WSSV infection were sampled. Approximate degrees of latitude and longitude are shown.

Samples of live tiger shrimp were collected from four different landing centres at locations around the coast of India (three individuals from Andamans: 11°40′N & 92°45′E; two from Chennai: 13°04′N & 80°17′E; two from Tuticorin: 8°48′N & 78°11′E; and three from Vishakhapatnam: 17°44′N & 83°23′E). About 200 tiger shrimp that survived a severe WSSV infection during pond culture at Bapatla (15°54′N & 80°30′E) in Andhra Pradesh were brought to the Central Institute of Brackishwater Aquaculture, Chennai and maintained. From this group, which survived for a further four months, three shrimp were sampled for the study. The Andamans exist as an island group on the eastern side of the mainland, whereas Chennai, Tuticorin and Vishakhapatnam are situated on the East coast (Figure 3). No specific permissions were required for these locations/activities since the shrimps were from a regular fishing ground, or from culture ponds, and not from a protected area, and since CIBA has a mandate from the Government of India to cover these areas for sampling. The field studies did not involve endangered or protected species since *P. monodon* does not come under these categories.

Shrimp were dissected under sterile conditions and fresh tissue samples (hepatopancreas, muscle, nervous tissue, intestinal tract, heart, gonad, gill, pleopod, eyestalk and lymphoid organ) were dissected out and stored in 1.5 ml microtubes. Microtubes were filled with sufficient RNAlater (Qiagen) to completely immerse the tissue sample, capped and stored at room temperature for two hours, 4°C overnight and −80°C until further use. Samples were sent to Genotypic Technology Pvt Ltd, Bangalore for RNA extraction.

### Preparation of RNA pools for RNA-seq

Table 5 shows individual shrimp and tissues that yielded high quality RNA and were used for subsequent sequencing. The RNA samples were pooled for subsequent sequencing in separate lanes of an Illumina Genome Analyser II flow cell. The first lane contained samples collected from the Andaman Islands (AI), the second lane contained samples from East coast locations in India (Chennai, Vishakhapatnam and Tuticorin, collectively referred to as EC) and the third contained samples from shrimp that survived a WSSV outbreak on a farm in Bapatla (SUR). Pools were created in a way that resulted in a relatively even contribution of RNA from each individual and tissue from the three zones. Equal quantities (by weight) of tissue were used to create 30 different tissue homogenates representing the 10 tissues (hepatopancreas, muscle, nervous tissue, intestinal tract, heart, gonad, gill, pleopod, eyestalk and lymphoid organ) from each of the three locations (AI, EC and SUR). After RNA extraction, equal quantities of purified RNA from the 10 tissue homogenates were pooled to create the final *AI*, *EC* and *SUR* RNA pools.

**Table 5:** Individuals, locations and tissues sampled for RNA-seq.

Individual	Location/designation	Hepatopancreas	Muscle	Nervous tissue	Intestinal tract	Heart	Testes	Ovary	Gills	Pleopod	Eyestalk	Lymphoid organ
1	Andaman	√	√	√	√	√	√		√			
2	Andaman	√		√		√	√		√	√		√
3	Andaman	v	√	√		√		√	√			v
4	Chennai	√	√	√	√	√	√		√	√		√
5	Chennai	√	√	√	√	√		√	√	√	√	√
6	Vishakhapatnam	√	√	√	√	√	√		√		√	√
7	Vishakhapatnam	√	√	√	√	√		√		√	√	√
8	Vishakhapatnam	√	√	√		√			√	√	√	√
9	Tuticorin	√	√	√	√	√		√	√	√	√	
10	Tuticorin	√	√	√	√	√		√	√	√	√	√
11	Survivor	√	√	√	√	√	√		√	√		√
12	Survivor		√	√	√	√	√		√	√	√	√
13	Survivor	√	√	√	√	√	√		√	√	√	√

RNA was extracted (TRIzol® Plus RNA Purification, Invitrogen) according to manufacturer's instructions. Tissue was ground in liquid nitrogen and 1.5 ml of TRIzol was added. RNA was further purified by chloroform extraction followed by precipitation using isopropanol and one wash with 70% ethanol. RNA was resuspended in 25 µl of RNase-free water. Only samples with optimal purity (OD260/280 between 1.8 and 2.2; OD260/230 between 1.0 and 2.4) and to a concentration >50 ng/µl but <2500 ng/µl, estimated using UV spectrophotometry (Nanodrop) and capillary electrophoresis (Agilent Bioanalyser), were utilized for sequencing. Samples were prepared using the Illumina protocol outlined in “Preparing Samples for Sequencing of mRNA” (Part# 1004898 Rev. A September 2008). mRNA was purified from total RNA using magnetic oligo(dT) beads and fragmented using divalent cations under elevated temperature. cDNA was synthesized from the fragmented mRNA using Superscript II (Invitrogen, USA), followed by 2^nd^ strand synthesis. The ends of the cDNA fragments were repaired and phosphorylated using Klenow, T4 DNA Polymerase and T4 Polynucleotide Kinase. An ‘A’ base was added to the 3′ end of the blunted fragments, followed by ligation of Illumina adapters via T-A mediated ligation. The ligated products were size selected by gel purification and then PCR amplified using Illumina primers. Library size and concentration were determined using capillary electrophoresis (Agilent Bioanalyzer, Agilent Technologies, USA). Fragments of an average size of 300 bp were purified for sequencing after adapter ligation, and a final enrichment of purified cDNA templates was performed using PCR as per the Illumina protocol. No RNA normalization was performed. Seventy-two cycles of paired-end sequencing was performed on a Genome Analyser II (Illumina, USA) according to manufacturer's instructions.

### Processing of the raw Illumina GAII data files

Residual adapter and poor quality sequences (quality score threshold of 10) were trimmed from the raw data using the FASTX toolkit (6 June 2011, http://hannonlab.cshl.edu/fastx_toolkit/commandline.html), and reads shorter than 20 bp were discarded. Fastq files were converted to fasta files and *Trimest* (EMBOSS, 6 June 2011, http://emboss.open-bio.org/wiki/Appdoc:Trimest) used to trim poly A/T tails from the sequence.

### *De novo* assembly and annotation

*De novo* transcriptome assembly using CLC Assembly Cell Software version 4.0 (CLC bio, 6 June 2011, http://www.clcbio.com/) was performed using sequence from each pool (lane) with default parameters and a minimum contig size of 100. Subsequently, contigs from each lane were assembled using Phrap software (version 1.090518, 6 June 2011, http://phrap.org) to produce a joint reference. *De novo* assembly using data from all lanes simultaneously was not performed due to data quantity limitations in the version of CLC Assembly Cell used. Sequence data was deposited in NCBI's Gene Expression Omnibus [47]. Basic Local Alignment Search Tool applications (BLAST) [48] and BLAST databases were downloaded from the National Centre for Biotechnology Information (NCBI, http://blast.ncbi.nlm.nih.gov/Blast.cgi, January 2010) and installed locally on a UNIX cluster. BLASTX was used to compare nucleotide sequences to the non-redundant protein database with a Karlin-Altshul cut off E-score of 0.001. The ten best matches below the cut off threshold were retrieved, and the results imported into a Blast2GO database [49]. Annotated transcript sequence data is accessible through the Transcription Shotgun Assembly database of NCBI (accession numbers JR196815 – JR235449, http://www.ncbi.nlm.nih.gov/Genbank).

### SNP discovery and allele frequency estimation

The ‘find_variations’ program in CLC Assembly Cell was used to identify SNPs and indels. A range of minimum minor allele frequency and allele count thresholds were tested (Table 2), and an overall putative SNP/indel count was estimated using the criteria of a minimum coverage of six, minimum minor allele frequency of 0.05, and minimum minor allele count of 3. SNP detection was performed for the joint dataset (Table 2) and each pool (lane) separately.

### Transcript coverage

Separate assemblies of the sequence were created for each of the three pools using the annotated *de novo* assembly of all sequences as a reference. The program “assembly_info” in CLC Assembly Cell was used to calculate average sequence coverage for each contig within each pool.

### Identification of microsatellite loci

Contig sequences were searched using PrimerPro (http://webdocs.cs.ualberta.ca/~yifeng/primerpro/, July 2011) which is a pipeline for microsatellite identification integrating the microsatellite identification tool MISA, primer design software Primer3 and NCBI BLAST software. First contig sequences were trimmed of distal stretches containing ambiguous bases (2 in a 5′ 40 bp window stretch). Mono-, di-, tri-, tetra-, penta- and hexanucleotide sequences repeated more than 12, 8, 6, 6, 5 and 5 times respectively were captured as microsatellites with potential application for mapping and genome scans. Mononucleotide repeats of A or T, which often occurred at the 3′ or 5′ ends of the contigs, were removed from the list. The criteria used for primer design included a GC clamp of 1 base and an optimum melting temperature of 55°C (minimum 50 and maximum 60°C).

### *P. monodon* mitochondrial transcriptome

The complete *P. monodon* mitochondrial genome) was downloaded from GenBank (NCBI). This was used as a reference sequence for contig alignment using CLC Assembly Cell Software (CLC bio, 6 June 2011, http://www.clcbio.com/).

### Shrimp genotyped to produce the SNP linkage map

Adult males and non-gravid female tiger shrimp from the wild were procured from the East coast of India, kept in the quarantine facility of the Muttukadu Experimental Station (MES) of Central Institute of Brackishwater Aquaculture, 35 km south of Chennai, and transferred to the maturation facility of the Crustacean Culture Division of MES for breeding trials. Each family was produced by pairing two females and a male together for mating in one tonne fibre re-inforced plastic tanks. The shrimp were fed on a diet consisting of squid and polychaete worms which facilitates maturation. From maturation trials, seven full-sibling families were produced. Parents were sampled and stored at −80°C for DNA extraction. The offspring from these families were cultured in separate hapas in a pond to a size of about 3 to 5 g in order to retain family identity. Offspring from each hapa were sampled and stored at −80°C for DNA extraction.

### SNP genotyping

A custom design Illumina iSelect SNP-array, containing 6000 candidate loci was developed using the SNPs identified in the *de novo* assembled transcriptome (Dataset S1). The SNPs chosen for the array had minor allele frequency greater than 0.33 and contig length greater than 200. SNPs suitable for the Infinium II Assay (A/G, A/C, T/G or T/C with Illumina assay design scores greater than 0.85 and with sufficient flanking sequence for the assay, www.illumina.com) were chosen. Only one SNP per contig was selected for the assay. Probe preparation was performed by Illumina.

Genomic DNA was purified from 1024 offspring belonging to 7 full-sibling families using the phenol/chloroform method as described by [50] with slight modifications. The quality of extracted DNA was checked on 2% agarose gel in 1× TBE buffer after electrophoresing at 50V for an hour. The purity of DNA was checked using OD values at 260 and 280 nm. Quantification was achieved using the OD value at 260 nm measured by a Nanodrop 2000C (Thermo Scientific). Samples were genotyped following standard protocols for the Illumina iSelect SNP-array.

After genotyping, data was imported into the Genotyping Module of Illumina's Genome Studio software (V2011.1). Initial clustering of 5459 SNPs that converted successfully to assays and that were included on the array was performed using automatic settings and recorded pedigree information. Once complete, individual SNPs displaying relatively high rates of missing genotypes and/or pedigree errors were more closely examined and, where possible, cluster patterns were manually corrected to better reflect the markers behaviour. Furthermore, only markers classified subjectively as SNPs (presenting typical diploid 3-cluster pattern with well-spaced theta values representing pure AA, AB and BB genotypes) or “multisite variants 3” (presenting three clusters but with a skewed theta distribution indicating a duplicated locus with a polymorphism present at just one duplicate with the other being fixed) were included for subsequent analysis.

### Genomic pedigree checks on the family material used for mapping

Initial examination of the genotyped data revealed possible errors in the documented pedigree, therefore the SNP data was used to confirm the full-sibling family structure of all the genotyped offspring. Specifically, pairwise relatedness was calculated using COANCESTRY [51] to identify clusters of individuals and offspring representing true full-sibling families. When a cluster contained only one parent and offspring, genotypes of the other parent were inferred using the observed offspring and parent genotypes. Following the establishment of this ‘genomic pedigree’, data was checked for Mendelian inheritance errors using a script written by one of the authors in R and SNPs or animals showing consistent errors were removed from further analysis. The full-sibling families identified were subsequently used for linkage map construction.

### Linkage map

The linkage map was constructed separately based on male and female segregation using genomic pedigree data as input for the analysis using the software TMAP (http://users.math.yale.edu/~dc597/tmap/, [52]). Initially, the program *phasing* was used to define the marker phases in each family in the pedigree. Subsequently, *pedmerge* was used to merge these multiple phase-known pedigrees into a single data file. *Grouping* was used to identify groups of linked markers, with the LOD threshold varied from 20 to 60 until the number of groups reflected the expected number of chromosomes. Finally, *tmap* was used to order the markers within each linkage group. Both sex-specific and sex-averaged linkage maps were generated. Graphics of the linkage groups were generated with MAPCHART software [53].

Goodness of fit G-tests were used to test for segregation distortion (proportions differing from segregation ratios expected with Mendelian inheritance) within families for each SNP (using a chisq.test function in R). A Bonferonni correction (based on the number of linkage groups examined, which was 44 for *P. monodon*) was applied to limit experiment-wide error rates associated with multiple testing [54].

### Recombination rates

The intervals between adjacent markers common to the male and female maps were compared to assess differences in recombination rates between the sexes. A pair-wise t-test was used to test for significance over and within linkage groups.

### Genome coverage

Genome coverage was estimated by the methods described in Chakravarti et al. [55] and Fishman et al. [56]. The average framework marker spacing (s) was calculated by dividing the summed length of all linkage groups by the number of intervals. Genome length (L) was estimated by adding 2s to the length of each linkage group to account for ends beyond the terminal markers. Genome coverage for the male and female maps, c, was estimated as the percentage of the genome within d cM of a framework marker as c = 1 −e ^−2dn/L^, where n is the number of framework markers [55].

## Supporting Information

Figure S1 **Consensus male (M), female (F) and sex averaged (Avg) transcribed gene linkage maps for** ***Penaeus monodon*** **.** SNP marker names (contig number followed by position in bp) are shown to the right of each linkage group while position (in Kosambi cM relative to the upper marker in the group) is shown to the left.
(ZIPX)

Dataset S1 **Six thousand SNPs selected for inclusion on the Illumina SNP chip.** Blast2go annotations are shown. *Locus name* consists of the contig ID and position in bp along the contig.
(XLSX)

Dataset S2 **Occurrence of detected microsatellite units showing number of repetitions (5–38 times).**
(XLSX)

Dataset S3 **Microsatellite repeat array, contig location, primer sequence and gene identification.** *C*, complex repeating units. *P*, perfect repeating units. *Start* and *end* indicate positions in bp of microsatellites along each contig.
(XLSX)

Dataset S4 **Allele frequency of mitochondrial DNA SNPs in Andaman Island (AI) and East Coast (EC) sampled individuals.** SNP position in each contig, and in the overall mtDNA reference sequence [57] is indicated in bp.
(XLSX)

Dataset S5 **Gene ontology coding (GO terms) for transcripts containing linkage mapped SNPs.**
(XLSX)

Dataset S6 **Consensus BLAST annotation for transcripts containing linkage mapped SNPs.**
(XLSX)

Dataset S7 **Goodness of fit tests (G-test) comparing observed progeny ratios for each SNP in each family to ratios expected with Mendelian inheritance.** Markers showing significant segregation distortion after Bonferroni correction (P<0.05) are shaded in red.
(XLSX)
