# Supplementary figures and images for: The Development of a High Density Linkage Map for Black Tiger Shrimp (Penaeus monodon) Based on cSNPs
Source: PLoS One. 2014 Jan 17;9(1):e85413. doi: 10.1371/journal.pone.0085413 (PMC3894980; doi:10.1371/journal.pone.0085413)

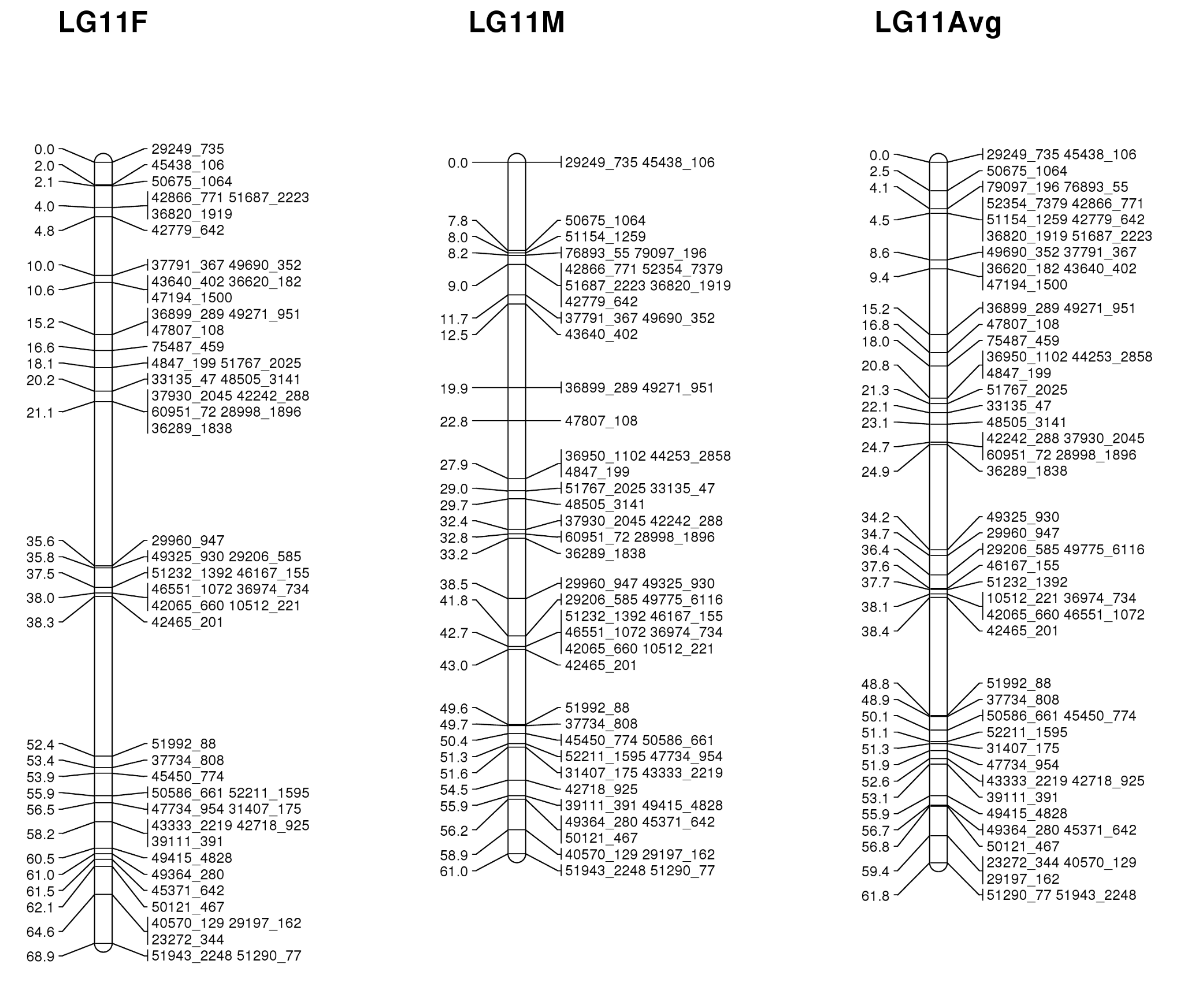

Supplement: Figure S1 — Consensus male (M), female (F) and sex averaged (Avg) transcribed gene linkage maps for Penaeus monodon . SNP marker names (contig number followed by position in bp) are shown to the right of each linkage group while position (in Kosambi cM relative to the upper marker in the group) is shown to the left. (ZIPX) [file pone.0085413.s001.zip › Figure S1 Linkage map 4_LG11.tif]

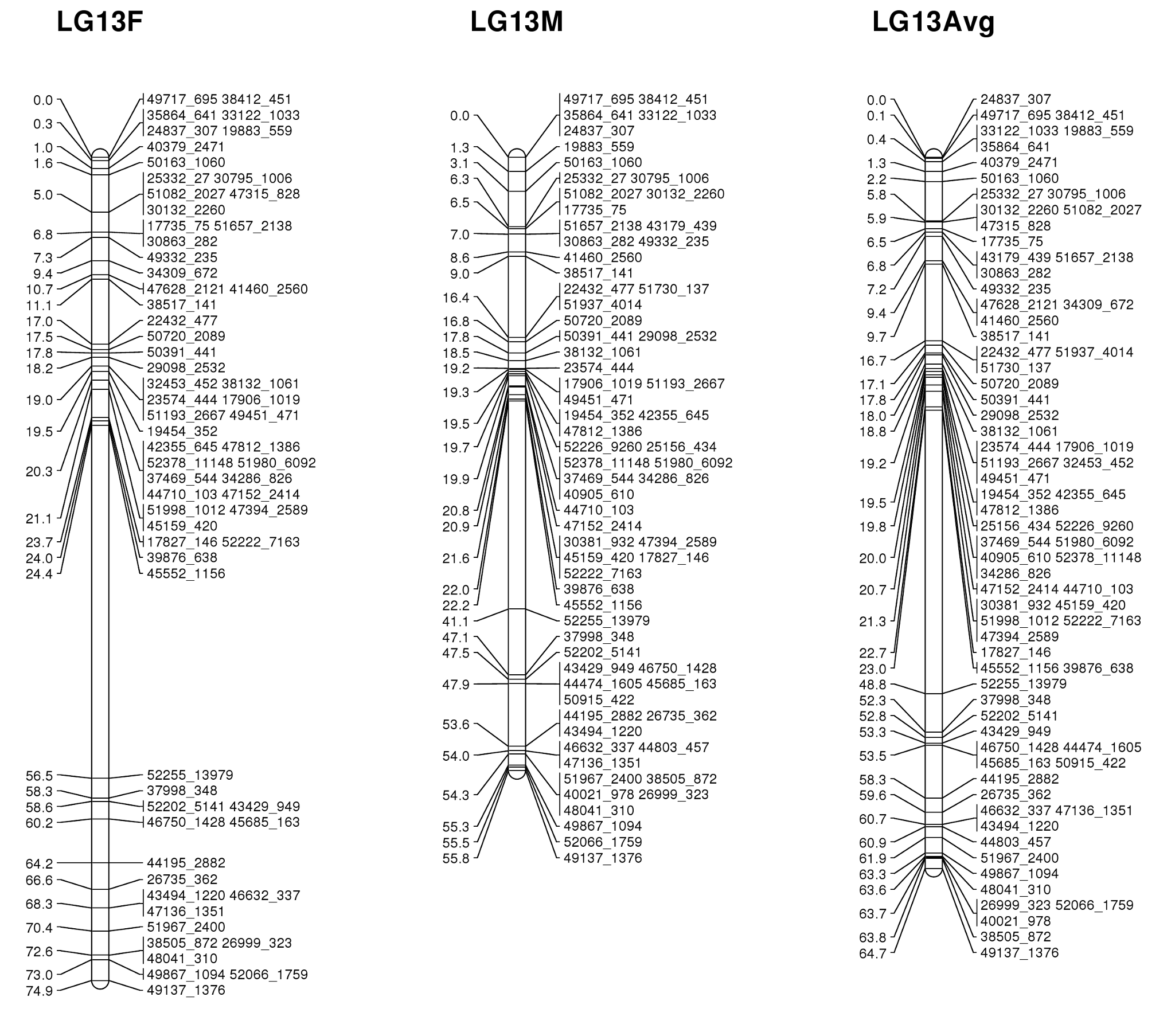

Supplement: Figure S1 — Consensus male (M), female (F) and sex averaged (Avg) transcribed gene linkage maps for Penaeus monodon . SNP marker names (contig number followed by position in bp) are shown to the right of each linkage group while position (in Kosambi cM relative to the upper marker in the group) is shown to the left. (ZIPX) [file pone.0085413.s001.zip › Figure S1 Linkage map 4_LG13.tif]

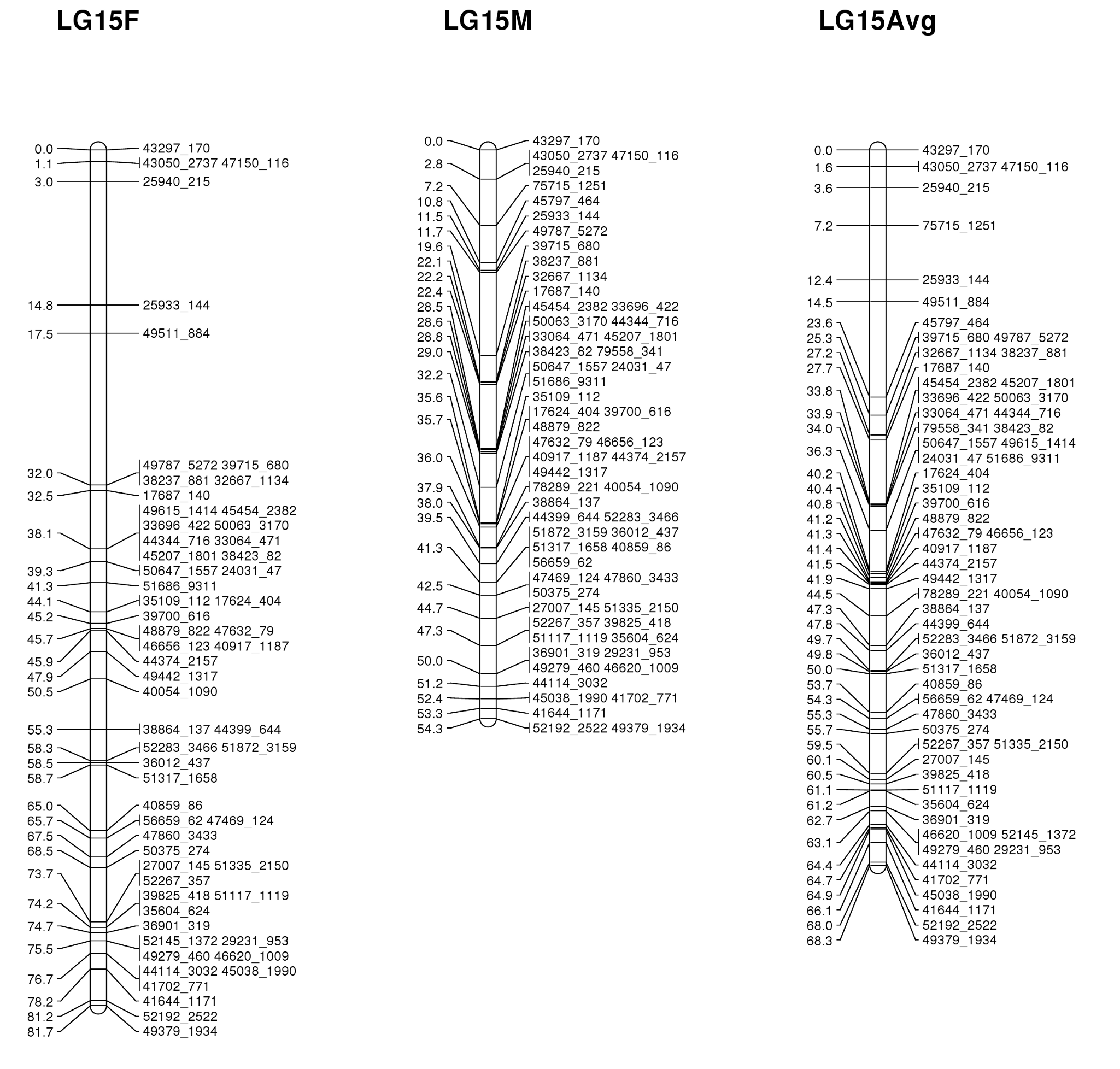

Supplement: Figure S1 — Consensus male (M), female (F) and sex averaged (Avg) transcribed gene linkage maps for Penaeus monodon . SNP marker names (contig number followed by position in bp) are shown to the right of each linkage group while position (in Kosambi cM relative to the upper marker in the group) is shown to the left. (ZIPX) [file pone.0085413.s001.zip › Figure S1 Linkage map 4_LG15.tif]

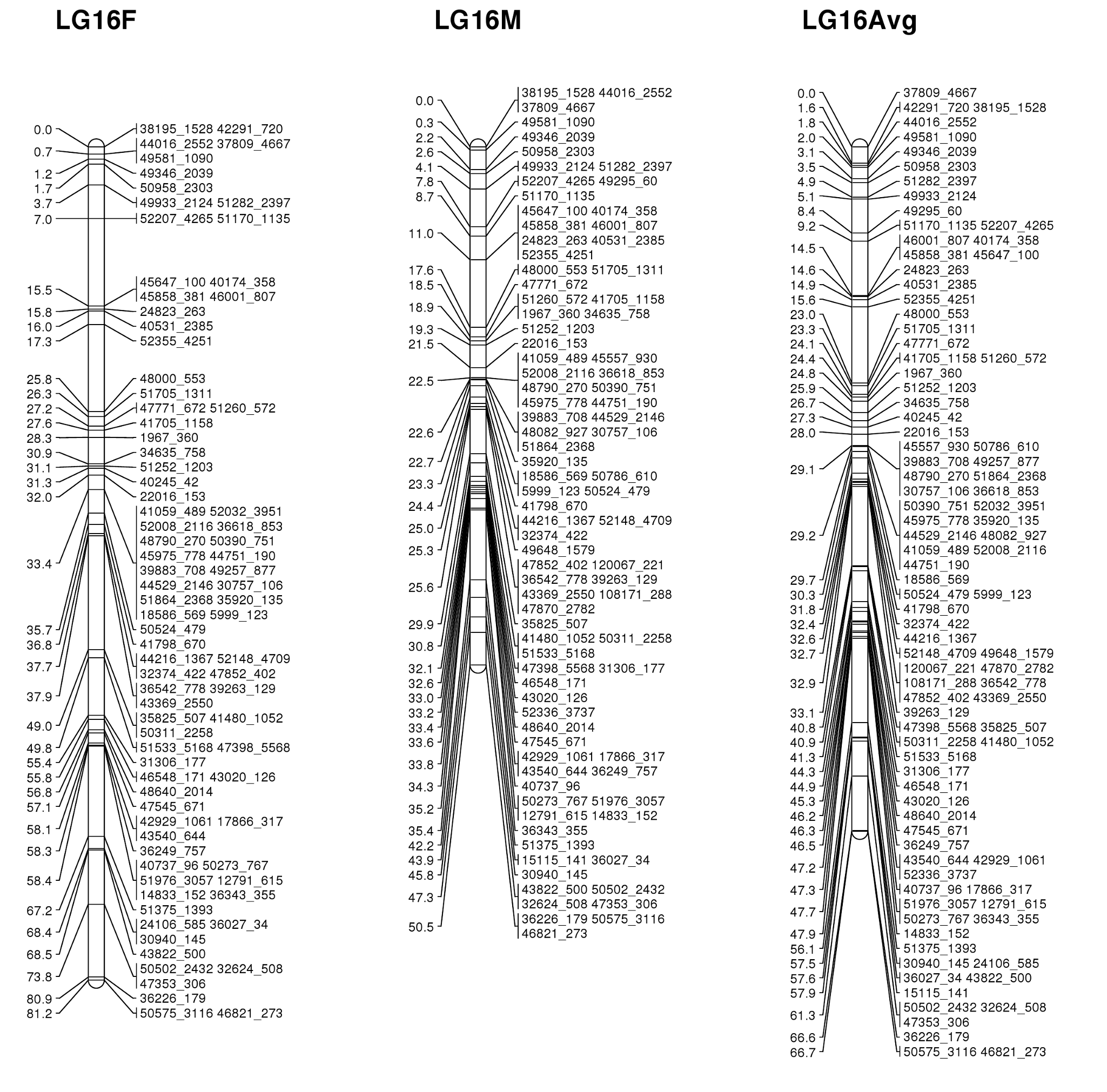

Supplement: Figure S1 — Consensus male (M), female (F) and sex averaged (Avg) transcribed gene linkage maps for Penaeus monodon . SNP marker names (contig number followed by position in bp) are shown to the right of each linkage group while position (in Kosambi cM relative to the upper marker in the group) is shown to the left. (ZIPX) [file pone.0085413.s001.zip › Figure S1 Linkage map 4_LG16.tif]

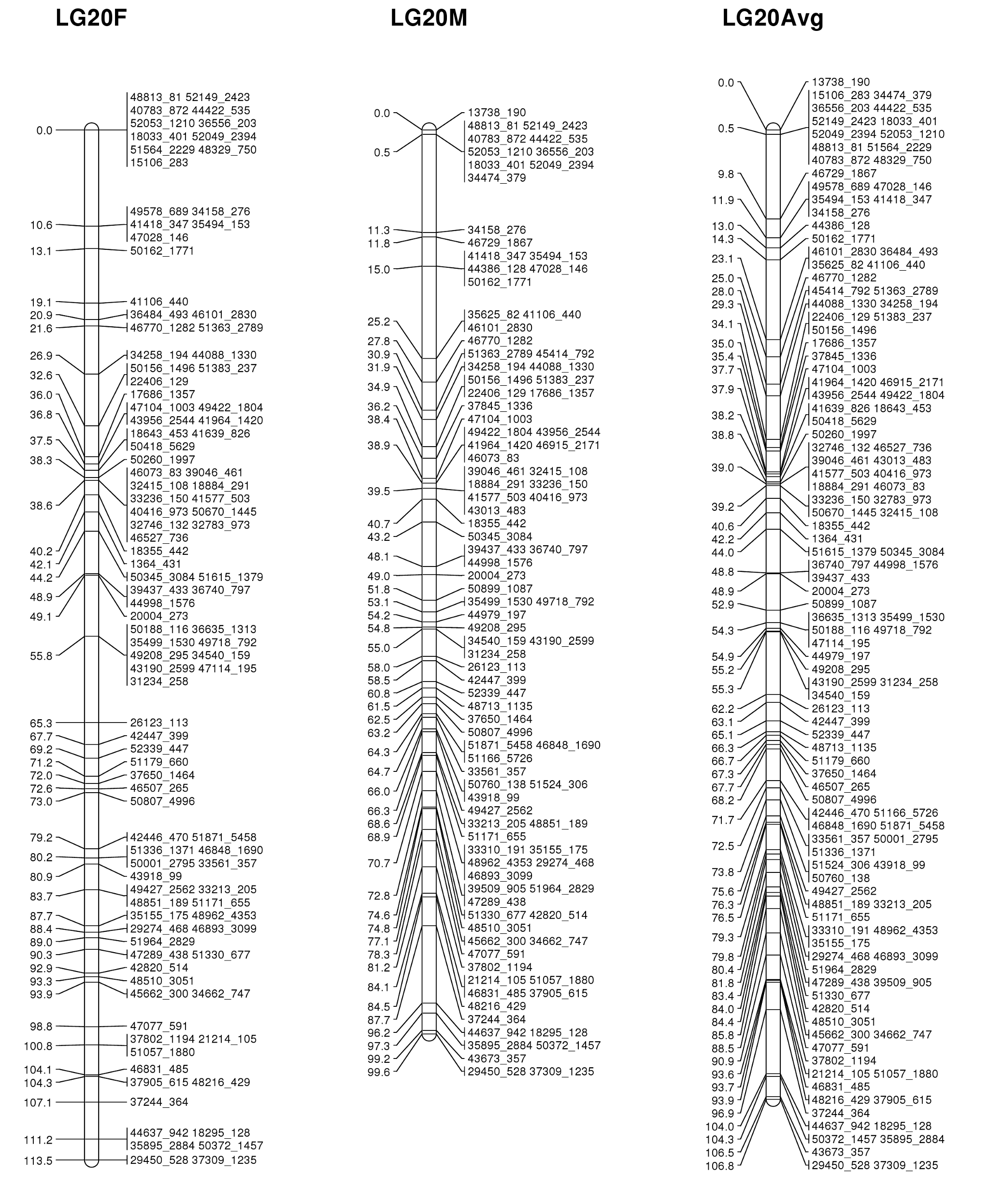

Supplement: Figure S1 — Consensus male (M), female (F) and sex averaged (Avg) transcribed gene linkage maps for Penaeus monodon . SNP marker names (contig number followed by position in bp) are shown to the right of each linkage group while position (in Kosambi cM relative to the upper marker in the group) is shown to the left. (ZIPX) [file pone.0085413.s001.zip › Figure S1 Linkage map 4_LG20.tif]

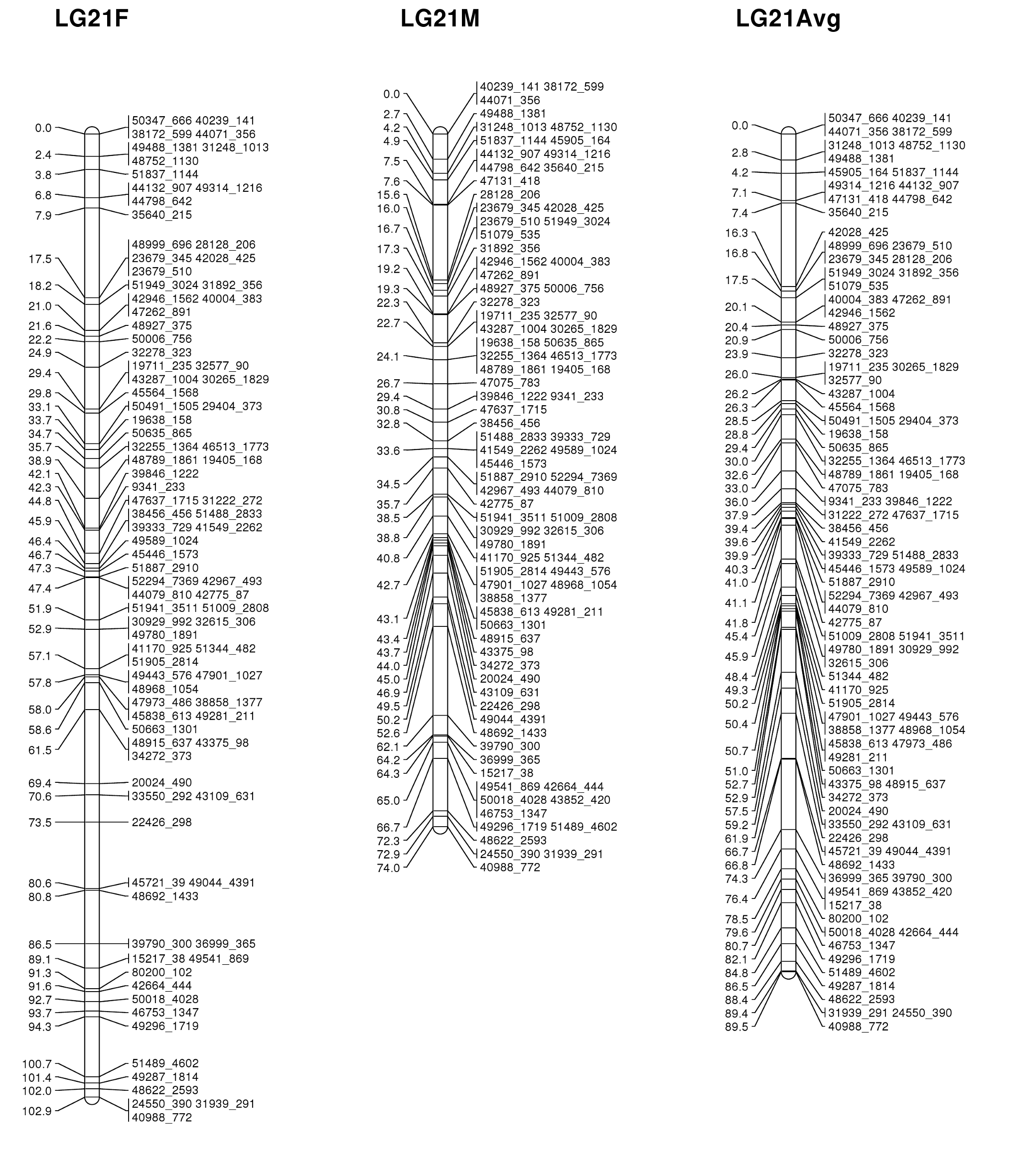

Supplement: Figure S1 — Consensus male (M), female (F) and sex averaged (Avg) transcribed gene linkage maps for Penaeus monodon . SNP marker names (contig number followed by position in bp) are shown to the right of each linkage group while position (in Kosambi cM relative to the upper marker in the group) is shown to the left. (ZIPX) [file pone.0085413.s001.zip › Figure S1 Linkage map 4_LG21.tif]

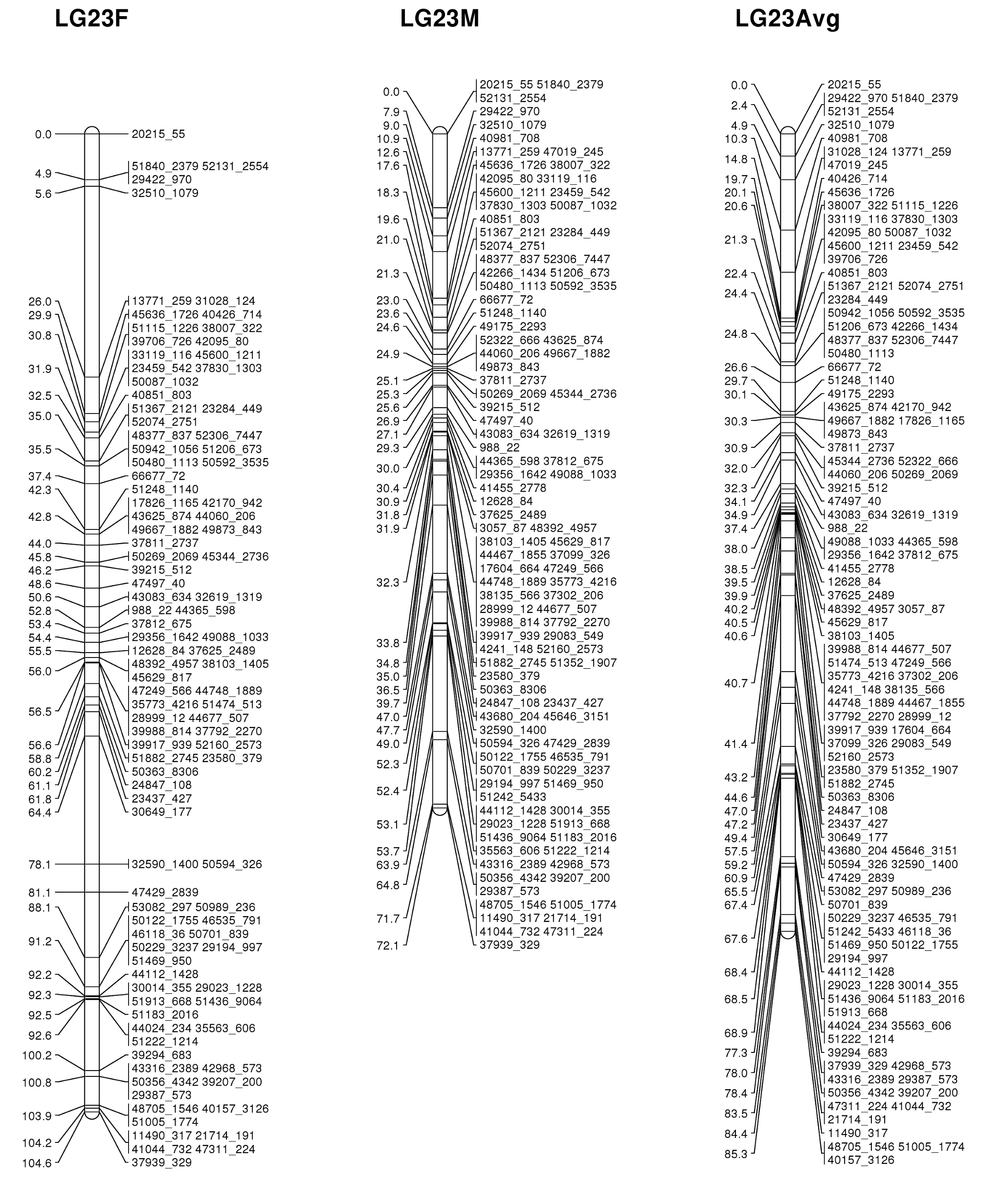

Supplement: Figure S1 — Consensus male (M), female (F) and sex averaged (Avg) transcribed gene linkage maps for Penaeus monodon . SNP marker names (contig number followed by position in bp) are shown to the right of each linkage group while position (in Kosambi cM relative to the upper marker in the group) is shown to the left. (ZIPX) [file pone.0085413.s001.zip › Figure S1 Linkage map 4_LG23.tif]

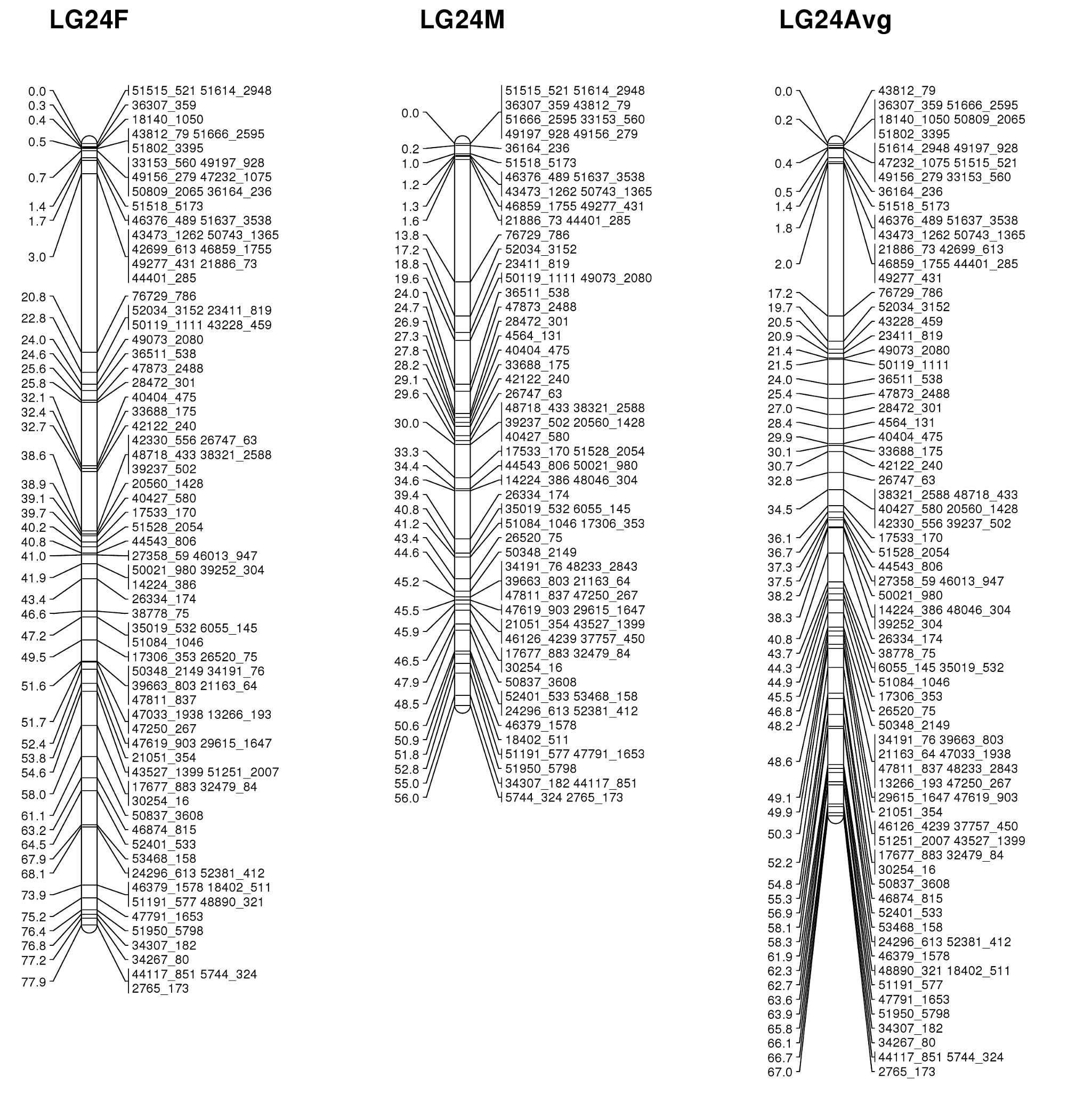

Supplement: Figure S1 — Consensus male (M), female (F) and sex averaged (Avg) transcribed gene linkage maps for Penaeus monodon . SNP marker names (contig number followed by position in bp) are shown to the right of each linkage group while position (in Kosambi cM relative to the upper marker in the group) is shown to the left. (ZIPX) [file pone.0085413.s001.zip › Figure S1 Linkage map 4_LG24.tif]

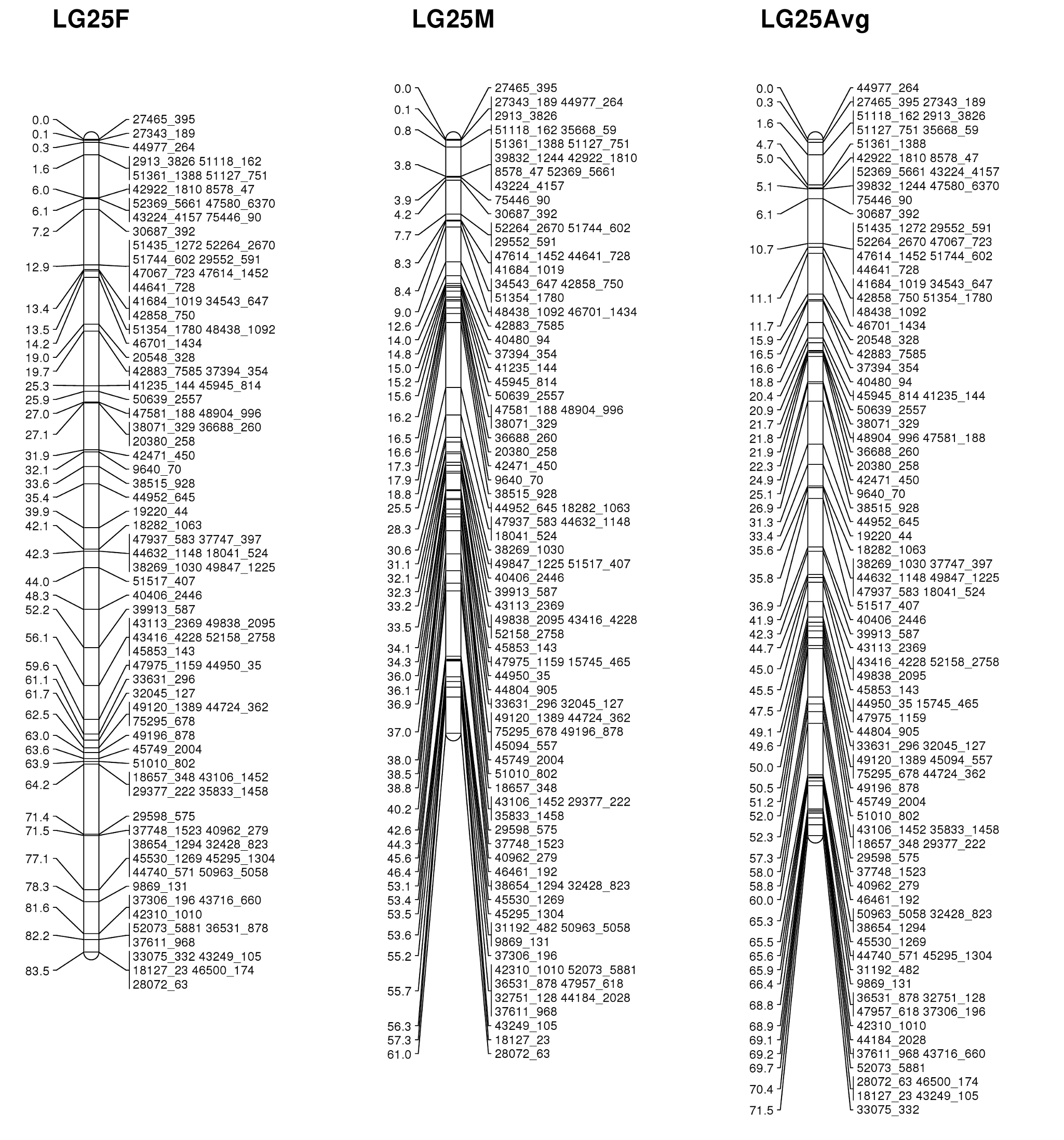

Supplement: Figure S1 — Consensus male (M), female (F) and sex averaged (Avg) transcribed gene linkage maps for Penaeus monodon . SNP marker names (contig number followed by position in bp) are shown to the right of each linkage group while position (in Kosambi cM relative to the upper marker in the group) is shown to the left. (ZIPX) [file pone.0085413.s001.zip › Figure S1 Linkage map 4_LG25.tif]

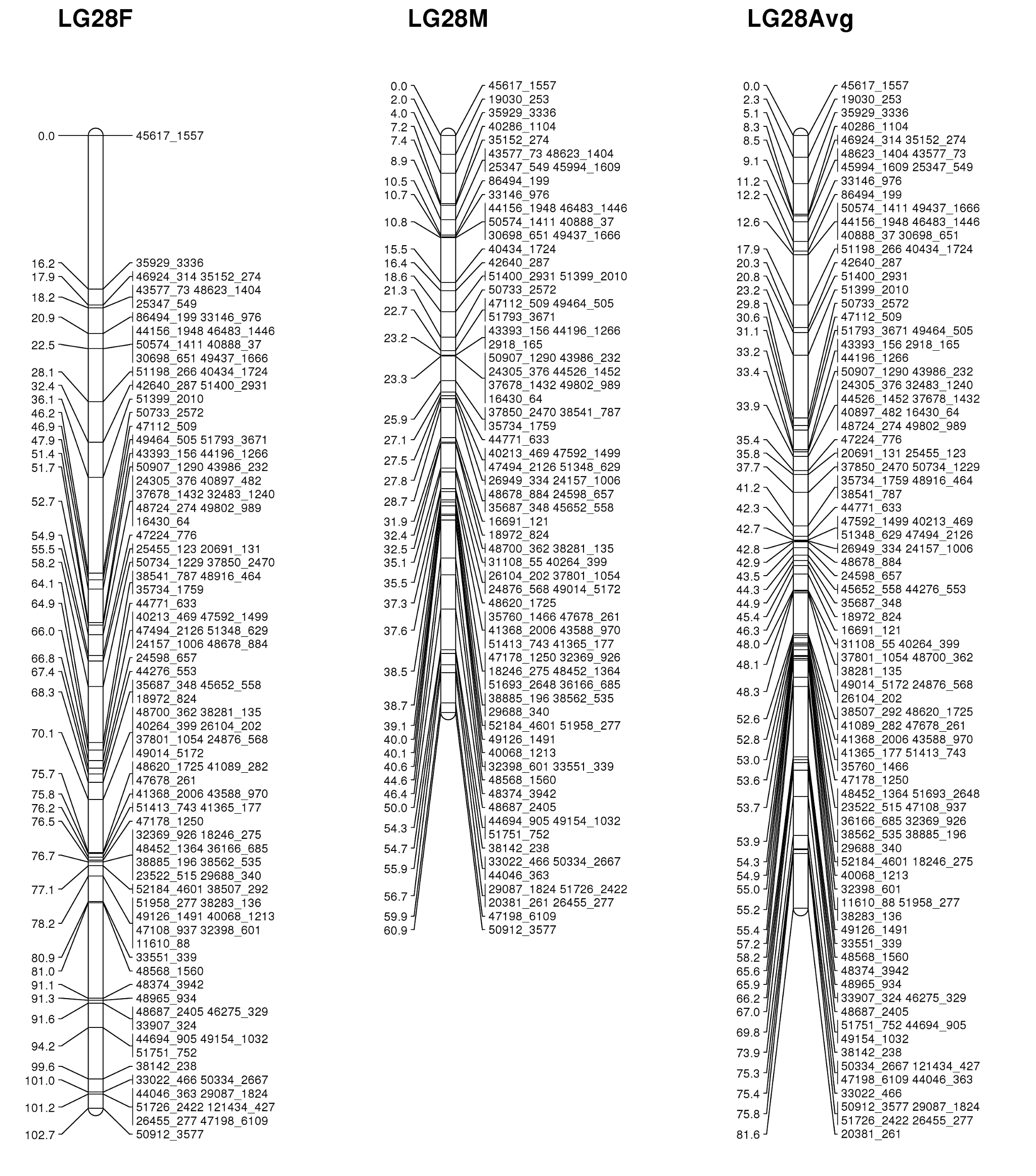

Supplement: Figure S1 — Consensus male (M), female (F) and sex averaged (Avg) transcribed gene linkage maps for Penaeus monodon . SNP marker names (contig number followed by position in bp) are shown to the right of each linkage group while position (in Kosambi cM relative to the upper marker in the group) is shown to the left. (ZIPX) [file pone.0085413.s001.zip › Figure S1 Linkage map 4_LG28.tif]

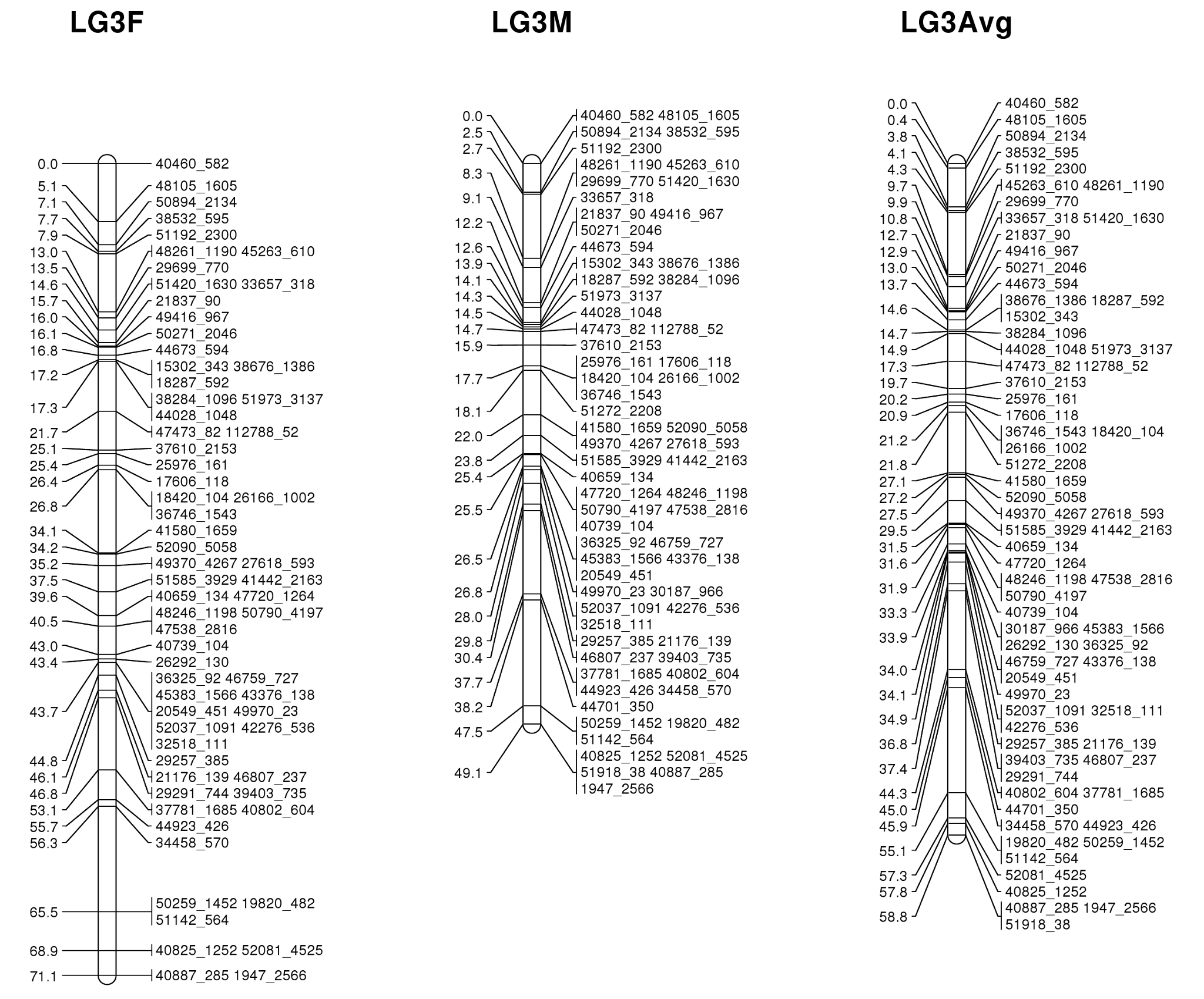

Supplement: Figure S1 — Consensus male (M), female (F) and sex averaged (Avg) transcribed gene linkage maps for Penaeus monodon . SNP marker names (contig number followed by position in bp) are shown to the right of each linkage group while position (in Kosambi cM relative to the upper marker in the group) is shown to the left. (ZIPX) [file pone.0085413.s001.zip › Figure S1 Linkage map 4_LG3.tif]

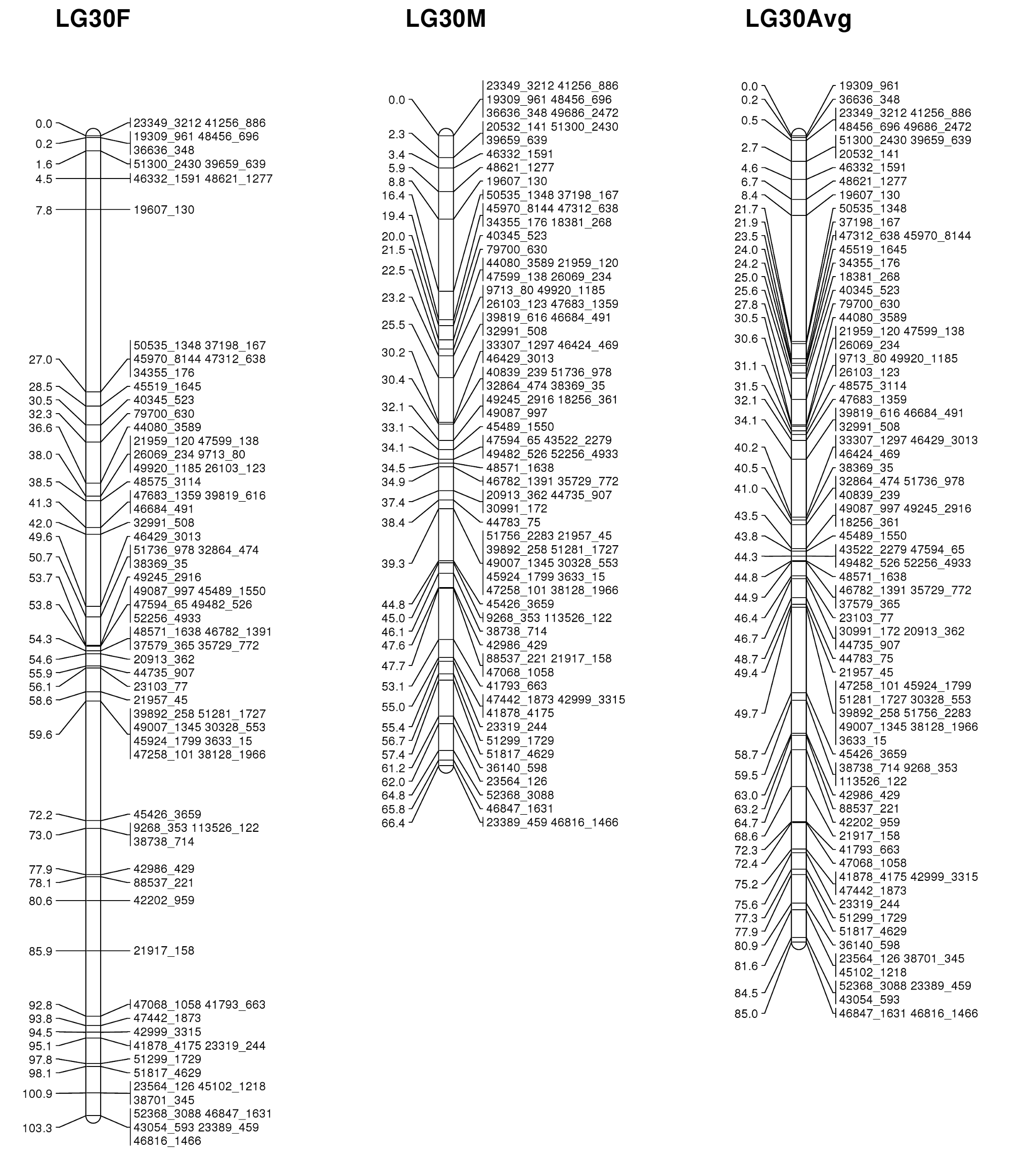

Supplement: Figure S1 — Consensus male (M), female (F) and sex averaged (Avg) transcribed gene linkage maps for Penaeus monodon . SNP marker names (contig number followed by position in bp) are shown to the right of each linkage group while position (in Kosambi cM relative to the upper marker in the group) is shown to the left. (ZIPX) [file pone.0085413.s001.zip › Figure S1 Linkage map 4_LG30.tif]

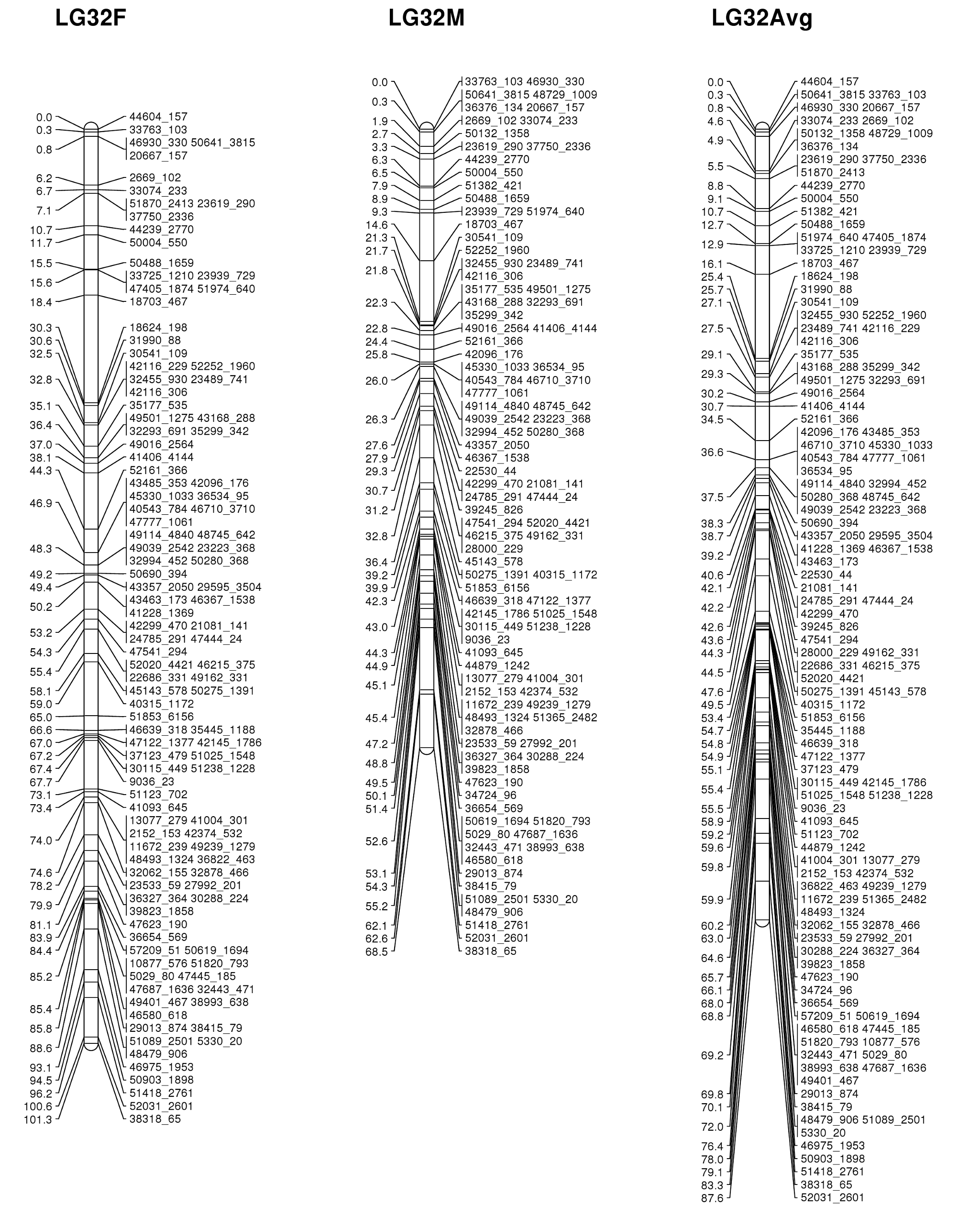

Supplement: Figure S1 — Consensus male (M), female (F) and sex averaged (Avg) transcribed gene linkage maps for Penaeus monodon . SNP marker names (contig number followed by position in bp) are shown to the right of each linkage group while position (in Kosambi cM relative to the upper marker in the group) is shown to the left. (ZIPX) [file pone.0085413.s001.zip › Figure S1 Linkage map 4_LG32.tif]

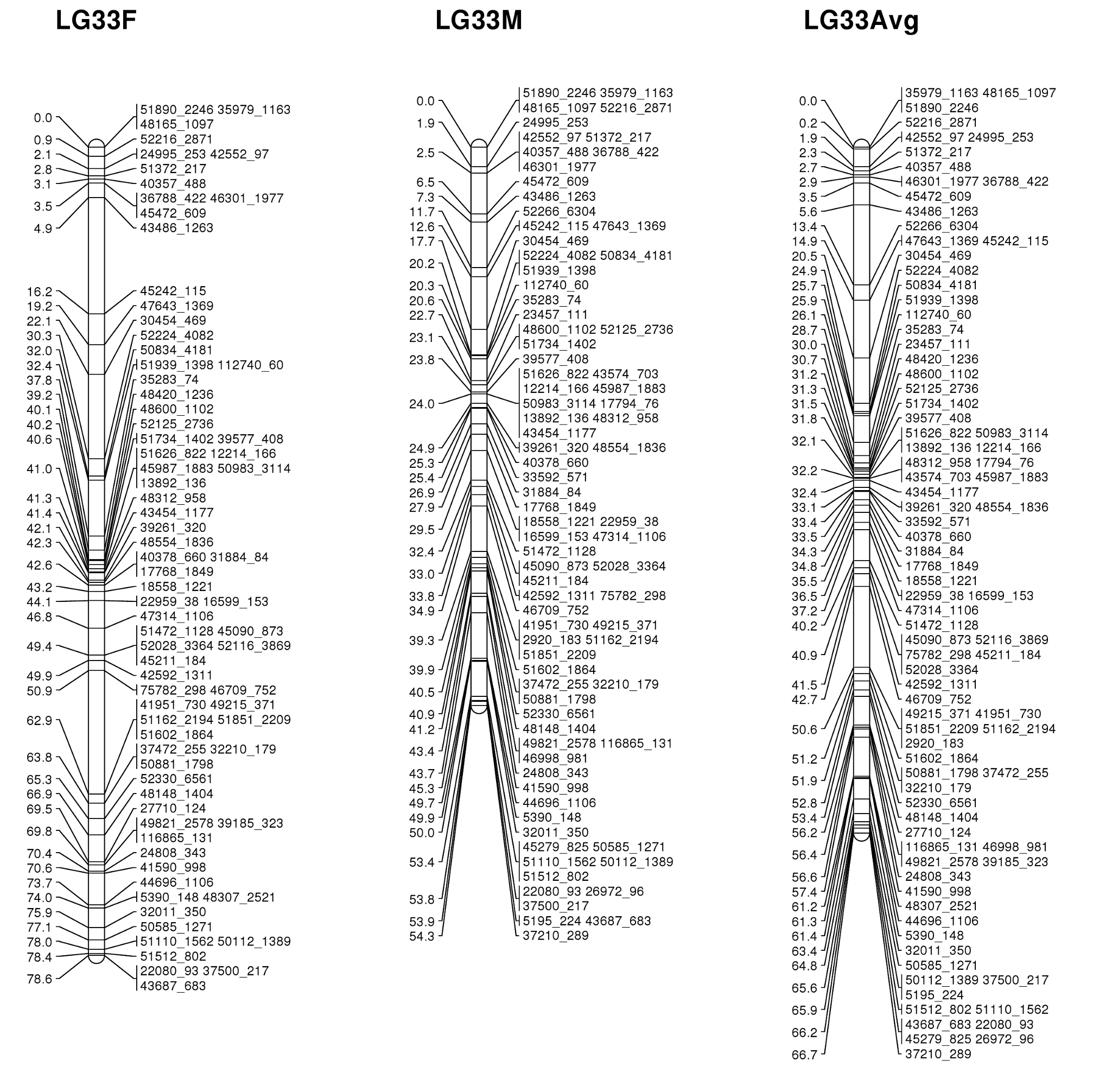

Supplement: Figure S1 — Consensus male (M), female (F) and sex averaged (Avg) transcribed gene linkage maps for Penaeus monodon . SNP marker names (contig number followed by position in bp) are shown to the right of each linkage group while position (in Kosambi cM relative to the upper marker in the group) is shown to the left. (ZIPX) [file pone.0085413.s001.zip › Figure S1 Linkage map 4_LG33.tiff]

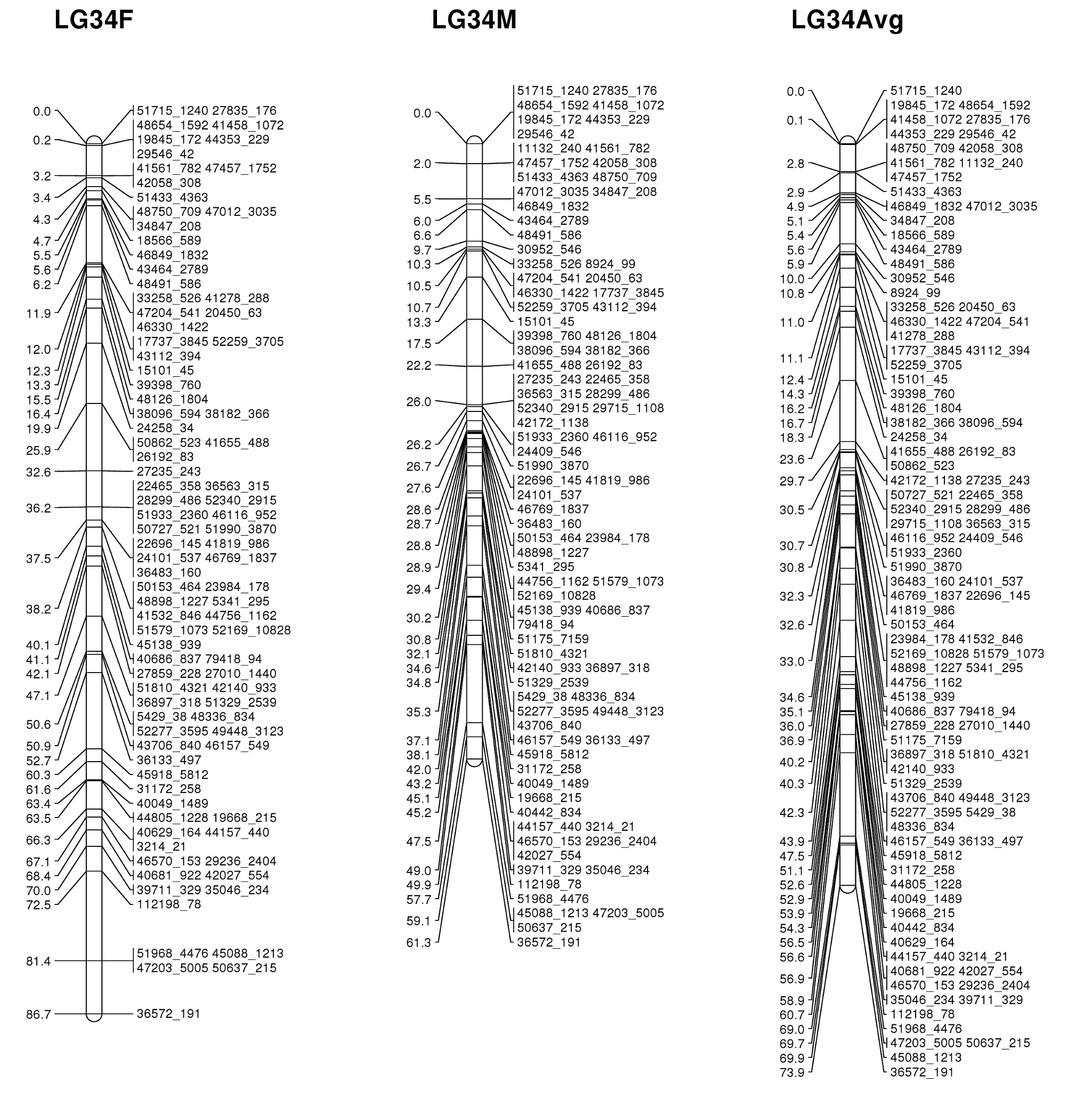

Supplement: Figure S1 — Consensus male (M), female (F) and sex averaged (Avg) transcribed gene linkage maps for Penaeus monodon . SNP marker names (contig number followed by position in bp) are shown to the right of each linkage group while position (in Kosambi cM relative to the upper marker in the group) is shown to the left. (ZIPX) [file pone.0085413.s001.zip › Figure S1 Linkage map 4_LG34.tif]

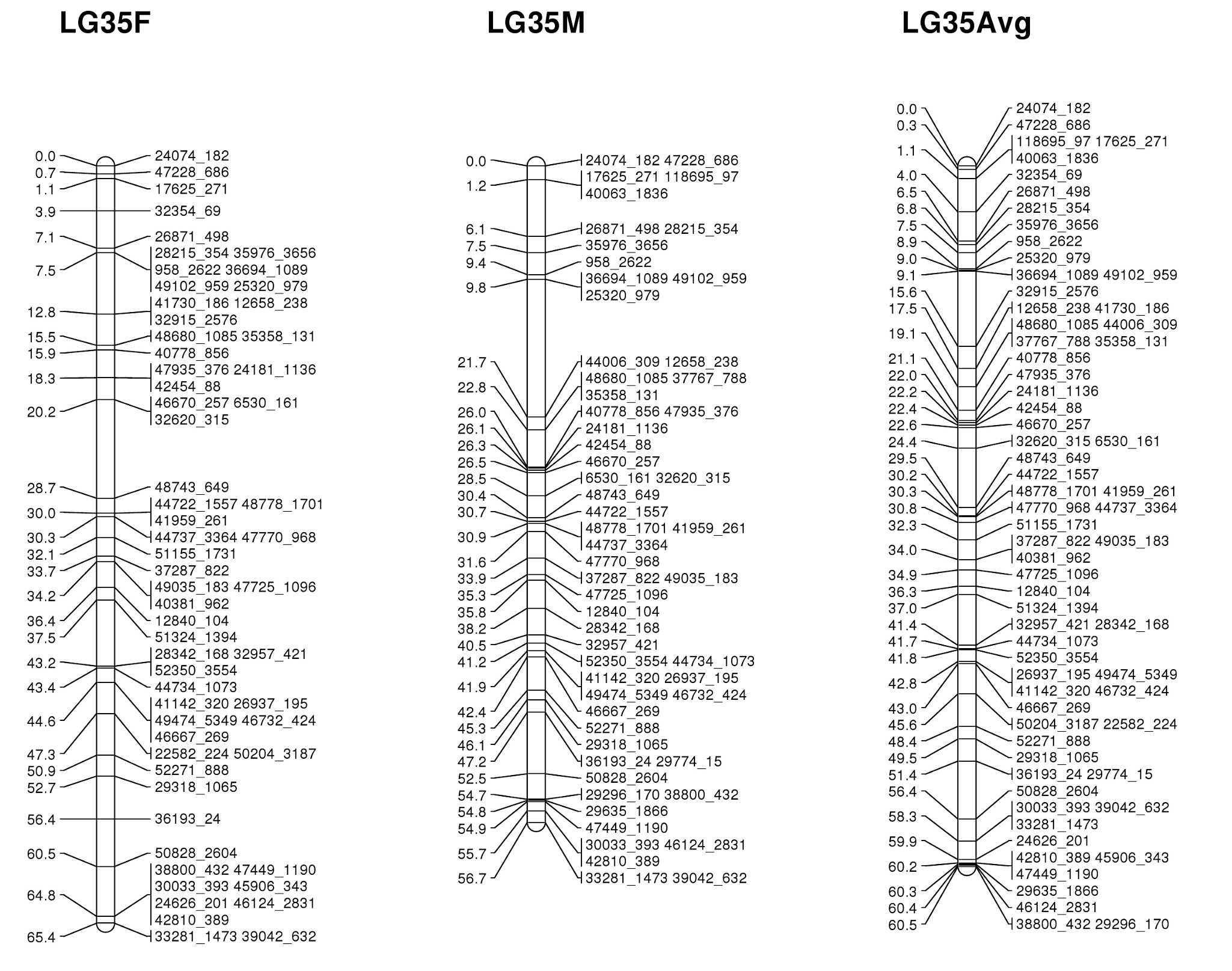

Supplement: Figure S1 — Consensus male (M), female (F) and sex averaged (Avg) transcribed gene linkage maps for Penaeus monodon . SNP marker names (contig number followed by position in bp) are shown to the right of each linkage group while position (in Kosambi cM relative to the upper marker in the group) is shown to the left. (ZIPX) [file pone.0085413.s001.zip › Figure S1 Linkage map 4_LG35.tif]

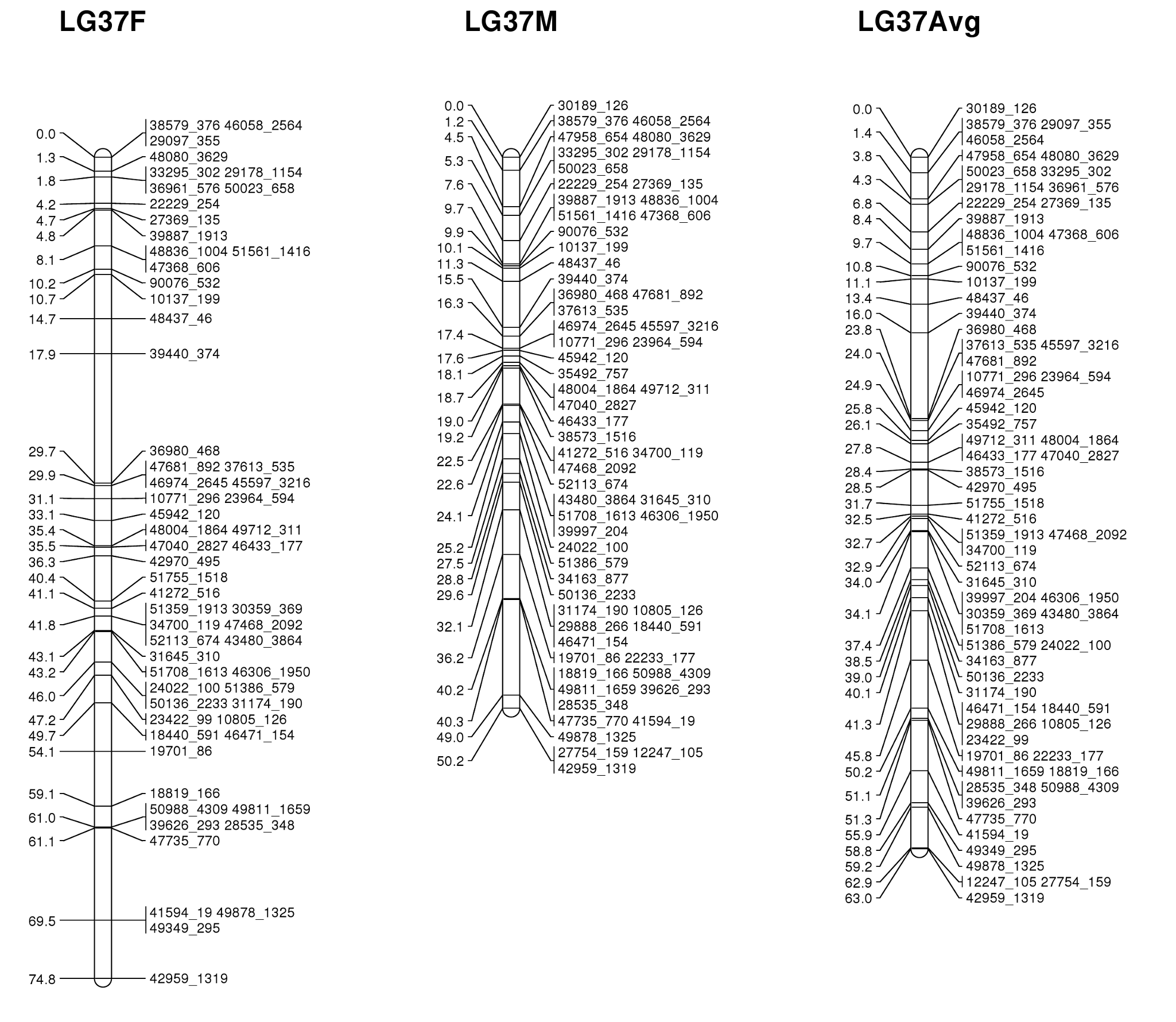

Supplement: Figure S1 — Consensus male (M), female (F) and sex averaged (Avg) transcribed gene linkage maps for Penaeus monodon . SNP marker names (contig number followed by position in bp) are shown to the right of each linkage group while position (in Kosambi cM relative to the upper marker in the group) is shown to the left. (ZIPX) [file pone.0085413.s001.zip › Figure S1 Linkage map 4_LG37.tif]

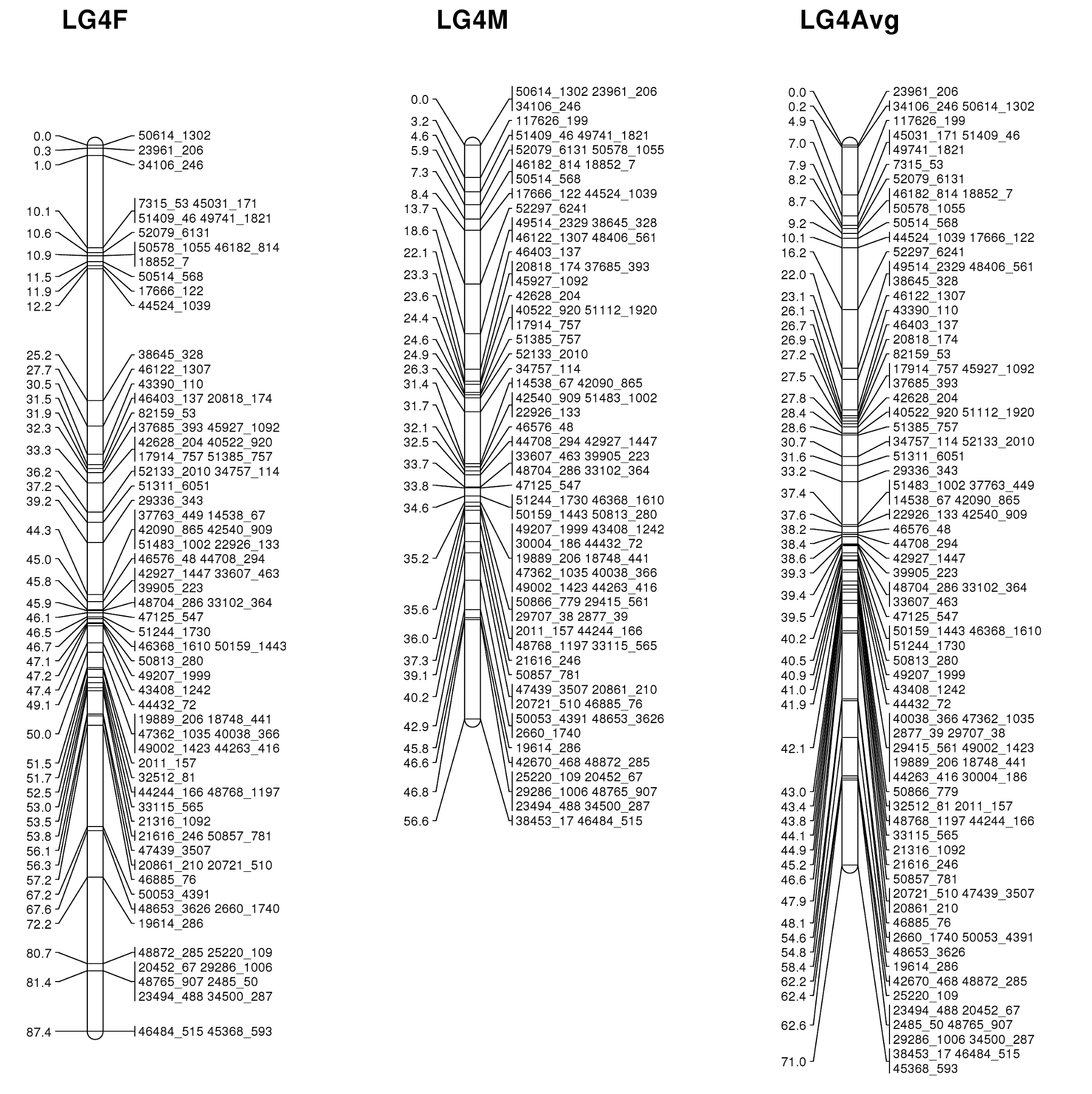

Supplement: Figure S1 — Consensus male (M), female (F) and sex averaged (Avg) transcribed gene linkage maps for Penaeus monodon . SNP marker names (contig number followed by position in bp) are shown to the right of each linkage group while position (in Kosambi cM relative to the upper marker in the group) is shown to the left. (ZIPX) [file pone.0085413.s001.zip › Figure S1 Linkage map 4_LG4.tif]

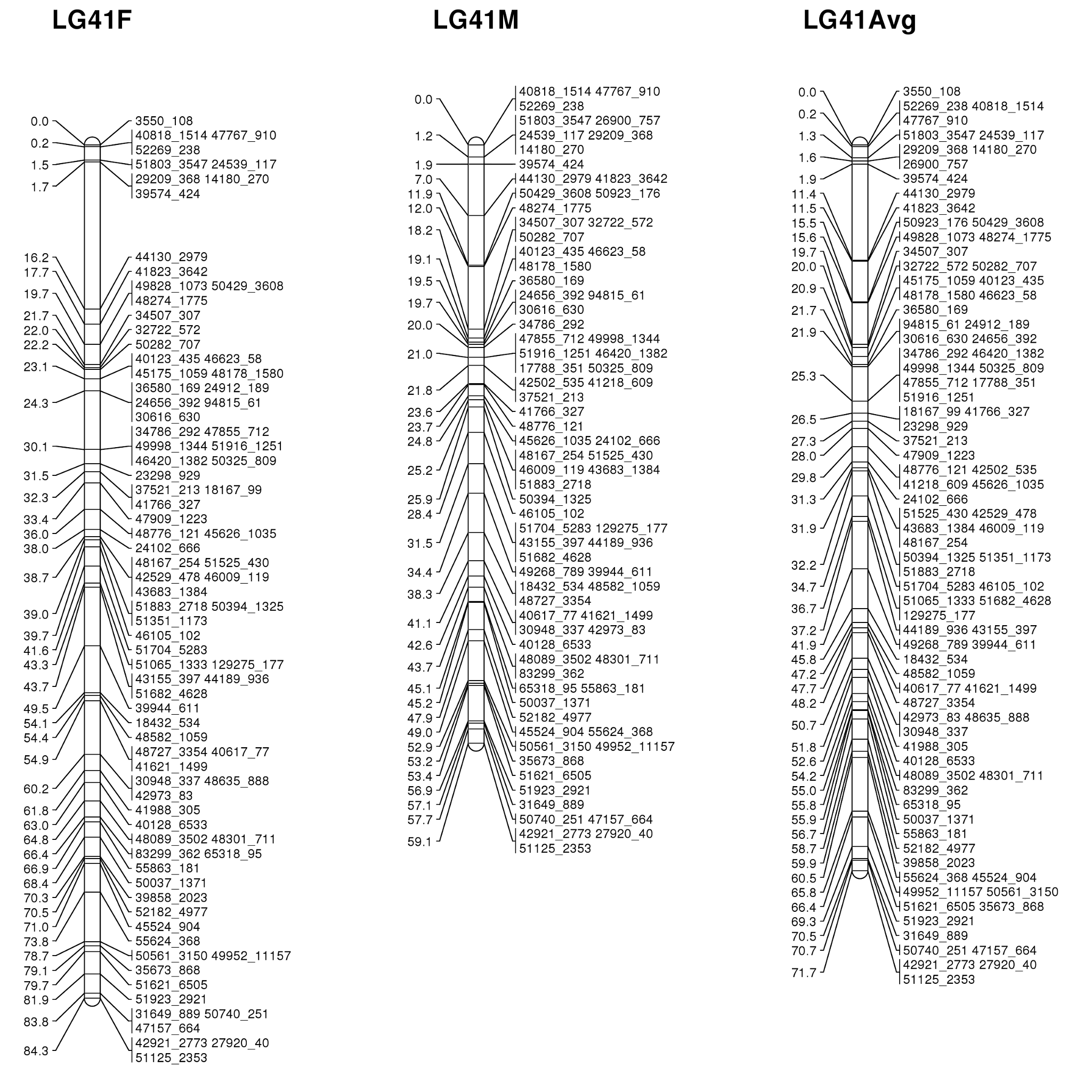

Supplement: Figure S1 — Consensus male (M), female (F) and sex averaged (Avg) transcribed gene linkage maps for Penaeus monodon . SNP marker names (contig number followed by position in bp) are shown to the right of each linkage group while position (in Kosambi cM relative to the upper marker in the group) is shown to the left. (ZIPX) [file pone.0085413.s001.zip › Figure S1 Linkage map 4_LG41.tif]

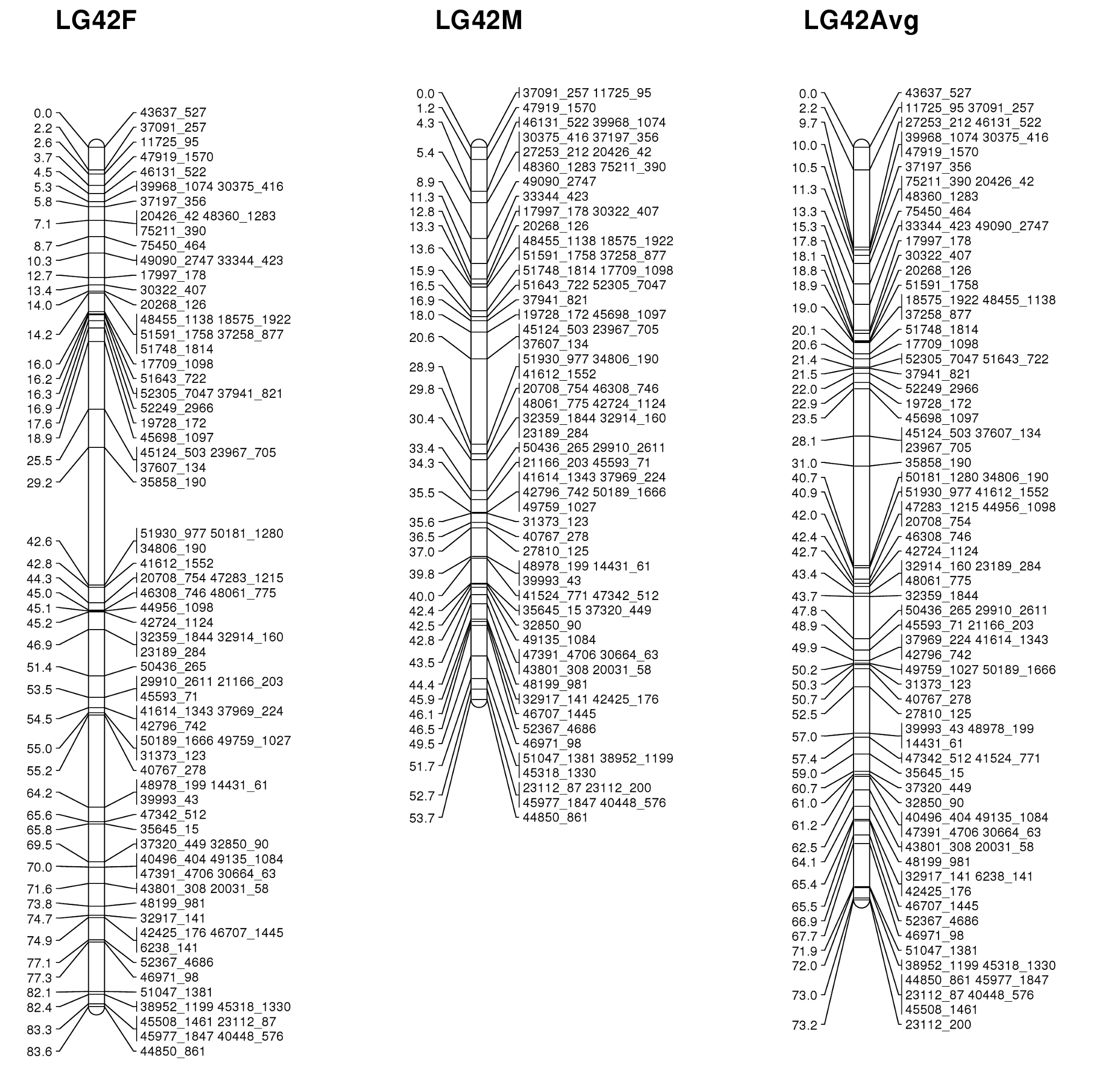

Supplement: Figure S1 — Consensus male (M), female (F) and sex averaged (Avg) transcribed gene linkage maps for Penaeus monodon . SNP marker names (contig number followed by position in bp) are shown to the right of each linkage group while position (in Kosambi cM relative to the upper marker in the group) is shown to the left. (ZIPX) [file pone.0085413.s001.zip › Figure S1 Linkage map 4_LG42.tif]

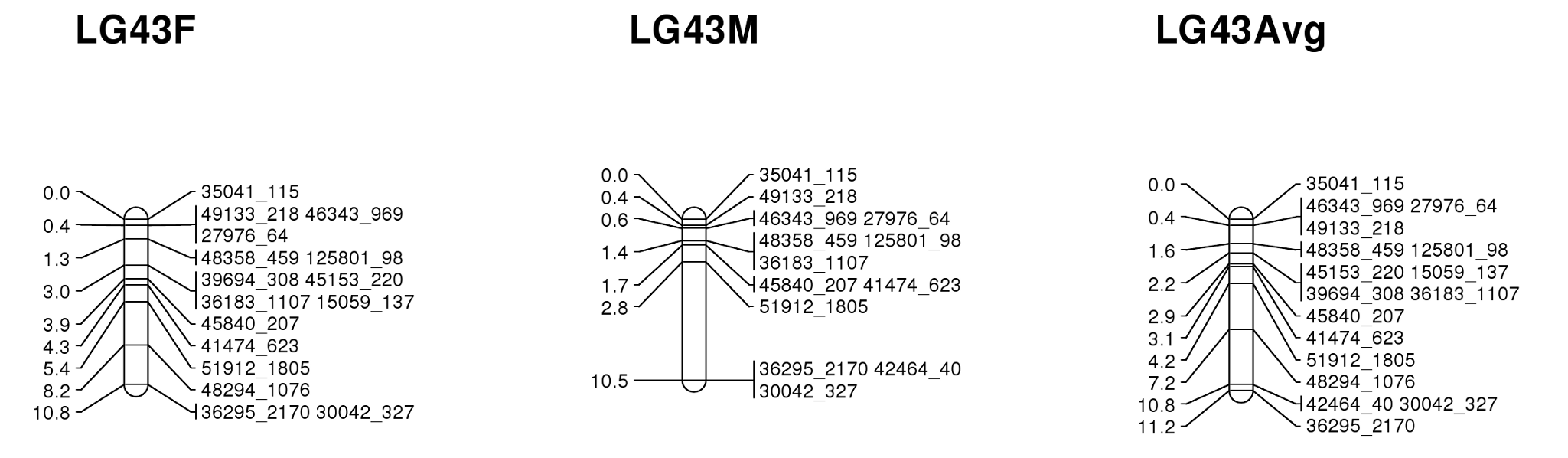

Supplement: Figure S1 — Consensus male (M), female (F) and sex averaged (Avg) transcribed gene linkage maps for Penaeus monodon . SNP marker names (contig number followed by position in bp) are shown to the right of each linkage group while position (in Kosambi cM relative to the upper marker in the group) is shown to the left. (ZIPX) [file pone.0085413.s001.zip › Figure S1 Linkage map 4_LG43.tif]

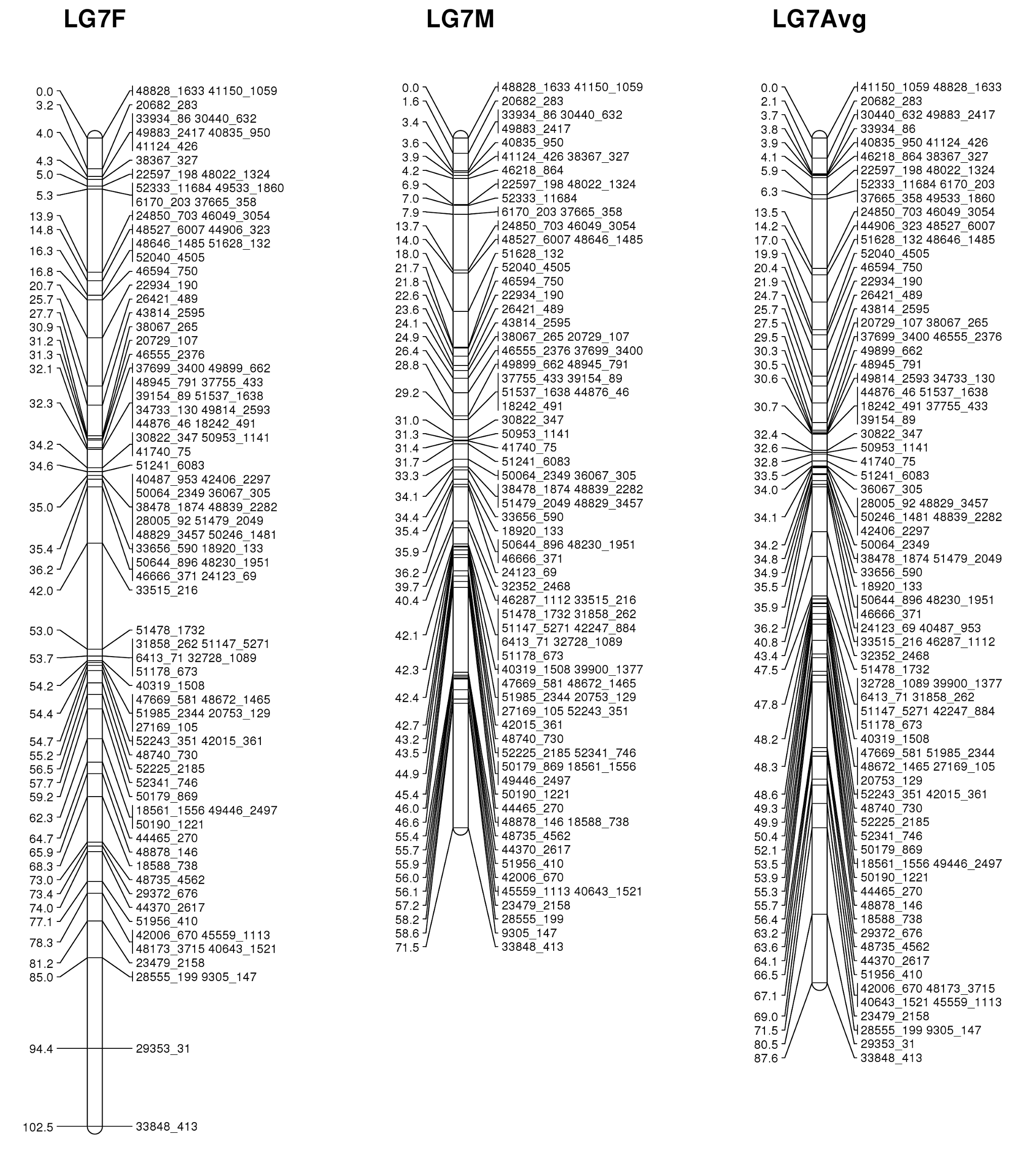

Supplement: Figure S1 — Consensus male (M), female (F) and sex averaged (Avg) transcribed gene linkage maps for Penaeus monodon . SNP marker names (contig number followed by position in bp) are shown to the right of each linkage group while position (in Kosambi cM relative to the upper marker in the group) is shown to the left. (ZIPX) [file pone.0085413.s001.zip › Figure S1 Linkage map 4_LG7.tif]

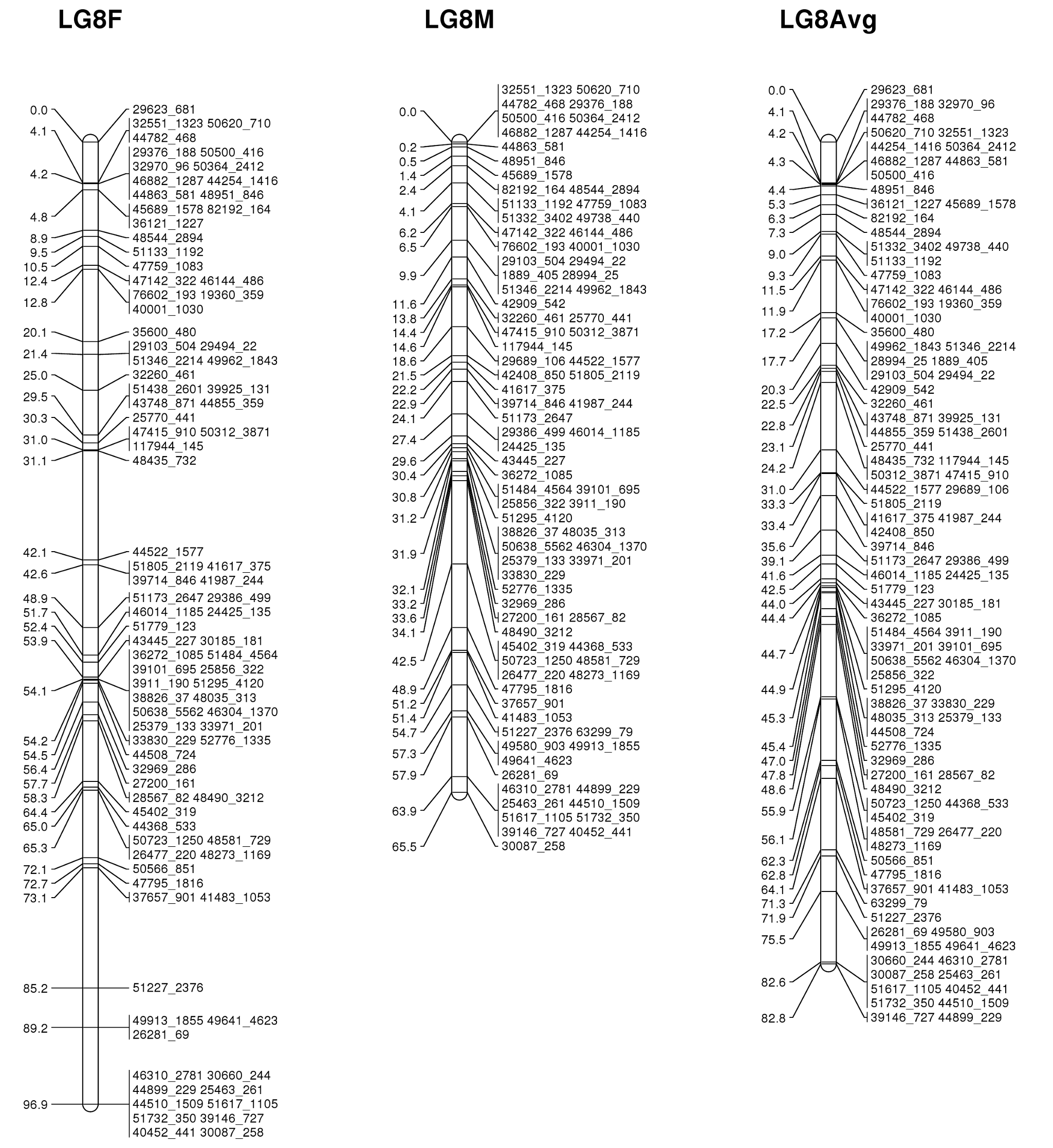

Supplement: Figure S1 — Consensus male (M), female (F) and sex averaged (Avg) transcribed gene linkage maps for Penaeus monodon . SNP marker names (contig number followed by position in bp) are shown to the right of each linkage group while position (in Kosambi cM relative to the upper marker in the group) is shown to the left. (ZIPX) [file pone.0085413.s001.zip › Figure S1 Linkage map 4_LG8.tif]
